# Psychometric validation of the Turkish version of the short nutrition literacy scale and its association with intuitive eating and body appreciation in young adults

**DOI:** 10.3389/fnut.2026.1780768

**Published:** 2026-07-08

**Authors:** Ezgi Makan, Salim Yilmaz, Ahmet Murat Gunal

**Affiliations:** 1 Department of Nutrition and Dietetics, Institute of Graduate Studies, Istanbul Okan University, Istanbul, Türkiye; 2 Department of Health Management, Faculty of Health Sciences, Graduate School of Health Sciences, Acibadem Mehmet Ali Aydinlar University, Istanbul, Türkiye; 3 Department of Nutrition and Dietetics, Faculty of Health Sciences, Halic University, Istanbul, Türkiye

**Keywords:** bifactor model, body appreciation, exploratory graph analysis, intuitive eating, measurement invariance, network psychometrics, nutrition literacy, scale validation

## Abstract

**Introduction:**

Nutrition literacy is a key determinant of dietary behaviors and long-term health outcomes, particularly during young adulthood, when lifelong eating patterns are established. Although the Short Nutrition Literacy Scale (S-NutLit) has been validated in several cultural contexts, evidence supporting a culturally appropriate and methodologically robust Turkish version remains limited. This study aimed to adapt and validate the Turkish version of the S-NutLit (S-NutLit-TR) and to examine its relationship with intuitive eating and body appreciation among Turkish young adults.

**Methods:**

This cross-sectional methodological study included 367 Turkish young adults aged 18–24 years. The S-NutLit was translated and culturally adapted following standardized guidelines. Dimensionality was examined using Exploratory Graph Analysis (EGA) and confirmed using confirmatory factor analysis and bifactor modeling. Measurement invariance across sex and body mass index (BMI) categories was also tested. Reliability was assessed using classical and bifactor indices. Construct validity was evaluated through correlations with intuitive eating and body appreciation. Additionally, mediation analyses examined whether intuitive eating mediated the relationship between nutrition literacy and body appreciation.

**Results:**

EGA revealed that SNUTLIT7 (knowledge of national dietary guidelines) exhibited critically low bootstrap stability (33.3%) and ambiguous dimensional assignment. Following its removal, structural consistency improved substantially (Expert Skills: 58.8% to 100%). The bifactor model demonstrated excellent fit (CFI = 0.997, RMSEA = 0.062), and the general factor explaining substantial common variance. Scalar invariance was achieved across sex and BMI categories. Nutrition literacy significantly predicted body appreciation through intuitive eating (β = 0.102), with expert skills showing full mediation via eating for physical rather than emotional reasons.

**Discussion:**

The 10-item S-NutLit-TR demonstrates robust psychometric properties and measurement equivalence across demographic subgroups. The integration of network psychometrics with bifactor modeling offers a rigorous framework for cross-cultural scale validation. The scale may serve as a valuable tool for nutritional epidemiology and public health interventions targeting young adults in Türkiye.

## 1 Introduction

Young adulthood represents a critical developmental period during which lifelong dietary habits are established, making it essential to equip individuals with adequate nutrition literacy skills during this formative stage. Nutrition literacy, an emerging area of research stemming from health literacy, has been defined as the ability to access, interpret, and use nutrition information (1). This construct is conceptualized as having three hierarchical levels: functional nutrition literacy, which relates to basic knowledge; interactive nutrition literacy, reflecting a person's ability to apply knowledge to changing circumstances; and critical nutrition literacy, which extends beyond knowledge and application to assess the ability to analyze nutrition information critically (1, 2). Food and nutrition literacy affects dietary behavior and nutritional intake, which subsequently leads to nutritional status disparities and further health impacts (3). Individuals with low nutrition literacy are at heightened risk for diet-related chronic diseases, including diabetes, hypertension, cardiovascular diseases, and stroke. Non-communicable diseases account for 41 million deaths annually, representing three-quarters of global mortality, with most of these conditions being largely preventable through adequate nutrition knowledge and healthy dietary patterns (4). Furthermore, malnutrition associated with inadequate nutrition literacy may manifest as both undernutrition and overnutrition. Undernutrition is linked to immune suppression, sarcopenia, and increased infection susceptibility, whereas overnutrition contributes to the global obesity epidemic that has nearly tripled in prevalence since 1975 (5). These consequences underscore the importance of improving nutrition literacy as an effective strategy to promote nutritional status, prevent chronic diseases, and enhance overall health outcomes.

In Türkiye, the assessment of nutrition literacy among young adults has gained increasing attention due to the rising prevalence of diet-related health problems. Nearly 60% of Turkish adults are classified as overweight or obese, and university students represent a particularly vulnerable population characterized by unhealthy dietary practices including low fruit and vegetable consumption, frequent fast-food intake, and irregular meal patterns (6). Despite this growing public health concern, validated instruments for assessing nutrition literacy in Turkish young adults remain limited.

The Short Nutrition Literacy (S-NutLit) scale, originally developed and validated by Vrinten et al. (7) among Dutch-speaking young adults in Belgium, represents a comprehensive yet brief instrument covering both information skills and expert skills domains. Although Koc et al. (8) subsequently translated and applied the S-NutLit in a Turkish sample, several limitations restrict the interpretability and generalizability of their findings, including a small sample size (*N* = 115) and recruitment from a single private university using convenience sampling. More importantly, the cultural adaptation of the scale was conceptually incomplete. In the Turkish version, the original reference to the “Flemish Food Triangle” was replaced with a generic item assessing knowledge of “the food triangle.” However, unlike the Belgian context—where the food triangle represents a nationally endorsed dietary framework—there is no officially recognized or widely disseminated food triangle model in Türkiye. National dietary guidance is instead provided through the Türkiye Dietary Guidelines (TÜBER), which do not adopt a triangular representation. This lack of a clear cultural referent introduces conceptual ambiguity and undermines content validity by shifting the item away from applied nutrition literacy toward vague or undefined knowledge.

These considerations highlight the need for a comprehensive cultural adaptation and re-validation of the S-NutLit for use in Türkiye. In addition, no previous Turkish study has examined the psychometric properties of the S-NutLit using advanced analytical approaches such as Exploratory Graph Analysis or bifactor modeling. Addressing these gaps, the present study aims to provide a methodologically rigorous and culturally appropriate validation of the S-NutLit-TR in a large and diverse sample of Turkish young adults.

Beyond its role in dietary behavior and chronic disease prevention, nutrition literacy has been increasingly linked to psychological constructs related to body image and eating behavior. Intuitive eating represents an adaptive eating pattern characterized by a strong connection with internal physiological hunger and satiety cues, whereby individuals are not preoccupied with food or dieting, do not label foods as “good” or “bad,” and often choose foods for the purpose of enhancing body functioning (9). This construct comprises four components: unconditional permission to eat, eating for physical rather than emotional reasons, reliance on hunger and satiety cues, and body–food choice congruence. Relatedly, body appreciation reflects individuals' favorable opinions of their bodies regardless of actual physical appearance, acceptance of the body despite perceived imperfections, respect for the body by attending to its needs, and protection of the body by rejecting unrealistic media images (10). Research has consistently demonstrated that intuitive eating is positively associated with body appreciation, self-esteem, and life satisfaction, whilst being inversely related to eating disorder symptomatology, body surveillance, body shame, and internalization of media appearance ideals (9, 10). In the Turkish context, Arslan et al. (11) demonstrated that food literacy serves as a significant mediator in the relationship between body image dissatisfaction and body mass index among Turkish adults (*N* = 1,178), with individuals expressing body satisfaction exhibiting higher food literacy scores. Furthermore, emerging evidence suggests that nutrition-related knowledge acquired through digital environments can influence eating behavior not only at the cognitive level but also at the psychosocial level, affecting body image perceptions and weight-related self-stigma (12). These findings suggest that nutrition literacy may be associated with adaptive eating attitudes and positive body image, potentially through underlying relational mechanisms, yet this mediational pathway has not been empirically examined using the S-NutLit scale.

The present study sought to provide a comprehensive psychometric evaluation of the Turkish version of the S-NutLit scale in a larger and more diverse sample of university students. The dimensional structure of the scale was examined using confirmatory factor analysis and bifactor modeling to assess whether a general nutrition literacy factor sufficiently accounts for item variance or whether domain-specific dimensions contribute additional explanatory value. The equivalence of the measurement model across sex and body mass index categories was also evaluated to determine whether the scale operates consistently across these subgroups. In addition, construct validity was examined through a mediation model in which nutrition literacy was hypothesized to be associated with body appreciation via intuitive eating, thereby testing whether higher nutrition literacy is associated with more adaptive eating attitudes and, indirectly, a more positive body image. This directional pathway was theoretically grounded in the assumption that nutrition literacy, as a cognitive and skill-based construct, may shape individuals' eating behaviors by influencing how they interpret and apply nutrition information. In turn, intuitive eating reflects a behavioral and attitudinal pattern that has been consistently associated with more positive body image outcomes. Accordingly, the proposed model assumes that nutrition literacy is related to body appreciation indirectly through its association with adaptive eating behaviors. By addressing methodological gaps in previous validation studies and situating the scale within a theoretically grounded nomological network, this study provides robust evidence supporting the use of the S-NutLit for assessing nutrition literacy among young adults in Türkiye.

## 2 Materials and methods

### 2.1 Study design, participants and data collection

This study employed a cross-sectional methodological design with two primary objectives: (i) to adapt and validate the Turkish version of the Short Nutrition Literacy Scale (S-NutLit) using contemporary psychometric approaches, and (ii) to examine the mediating role of intuitive eating in the relationship between nutrition literacy and body appreciation among young adults.

The target population comprised young adults aged 18–24 years residing in Türkiye. The lower age limit of 18 years was determined to ensure legal capacity to provide informed consent. Although the original S-NutLit validation study (7) included individuals aged 18–25 years, no explicit methodological justification for the upper age limit was provided in that study. Therefore, to ground the age range in an internationally recognized framework, the upper age limit in the present study was defined according to the United Nations statistical definition of youth, which classifies individuals aged 15–24 years as young people (13). This approach ensured conceptual consistency with global demographic standards while maintaining close alignment with the age range of the original validation study (7).

Data were collected between June 2024 and October 2025 through an online questionnaire hosted on Google Forms. Participants were recruited using snowball sampling via a multi-modal approach: (a) face-to-face recruitment using QR codes linking to the survey, and (b) direct links shared through WhatsApp. Prior to participation, all respondents provided informed consent by agreeing to the online consent form. The questionnaire took approximately 10–15 minutes to complete. Inclusion criteria were: (a) being aged 18–24 years, (b) residing in Türkiye, and (c) being able to read and understand Turkish. Exclusion criteria were: (a) being a dietitian or nutrition and dietetics student, as their professional training would naturally inflate nutrition literacy scores, and (b) incomplete or invalid responses.

Sample size considerations were guided by psychometric validation requirements. For scales with fewer than 20 items, a minimum of 200 participants is recommended to ensure stable factor solutions and reliable parameter estimates (14). Furthermore, the gold standard in scale validation involves conducting exploratory and confirmatory factor analyses on separate samples to avoid circular reasoning—where a structure derived from the data is “confirmed” using the same data (15). However, when total sample size is limited, this split-sample approach may compromise statistical power in each subsample.

**Figure 1:**
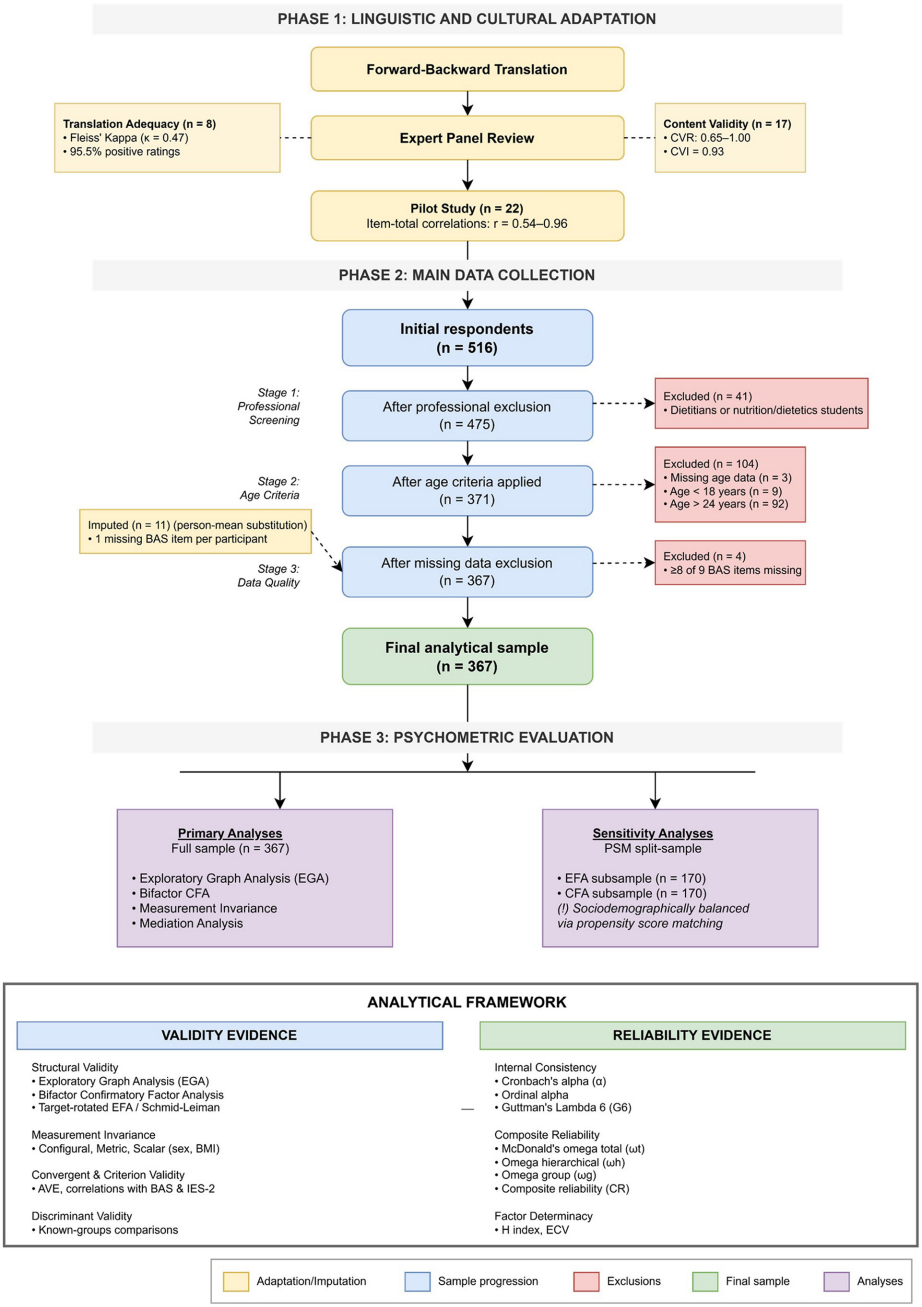
Participant flow and analytical design.

To address this methodological challenge, we employed a dual analytical strategy. First, primary analyses (Exploratory Graph Analysis, bifactor CFA, and measurement invariance testing) were conducted on the full sample (*n* = 367) to maximize statistical power. Second, as a sensitivity analysis, propensity score matching (PSM) was used to create two sociodemographically balanced subsamples (*n* = 170 each) for independent EFA and CFA, ensuring that any differences between subsamples reflected true structural variations rather than demographic confounds. Additionally, measurement invariance was tested across sex and BMI categories to confirm that the scale provided equivalent measurements across subgroups. This comprehensive approach strengthened the validity evidence while acknowledging sample size constraints (Figure 1).

### 2.2 Instruments

The questionnaire consisted of four sections: sociodemographic and anthropometric information, the Body Appreciation Scale (BAS), the Intuitive Eating Scale-2 (IES-2), and the Short Nutrition Literacy Scale (S-NutLit). Permission to adapt the S-NutLit into Turkish was obtained from the original scale developers via email (C. Matthys, personal communication, January 18, 2024). Permission to use the validated Turkish versions of the IES-2 and BAS was obtained from the Turkish adaptation authors (Ü. Akirmak, personal communication, January 28, 2024; O. Bakalim, personal communication, January 29, 2024).

#### 2.2.1 Sociodemographic and anthropometric data

The first section collected participants' anthropometric measurements (self-reported height and weight), sociodemographic characteristics (age, sex, education level, perceived income status), health-related information (presence of chronic disease, medical nutrition therapy status), dietary habits (number of daily meals, previous nutrition education), and physical activity level. Body mass index (BMI) was calculated from self-reported height and weight and categorized according to World Health Organization criteria (16).

#### 2.2.2 Short nutrition literacy scale (S-NutLit)

The S-NutLit was developed by Vrinten et al. (7) to assess nutrition literacy among young adults aged 18–25 years. The original scale comprises 11 items measuring two dimensions: Information Skills (8 items; items 1–8) assessing abilities to access, apply, and evaluate nutrition information, and Expert Skills (3 items; items 9–11) assessing the use of expert and scientific information sources. Items 1, 9, 10, and 11 use a frequency response format (1 = Never to 5 = Always), while the remaining items use an agreement format (1 = Strongly disagree to 5 = Strongly agree). Higher mean scores indicate greater nutrition literacy. The original scale demonstrated adequate internal consistency (Cronbach's α = 0.79–0.83). The S-NutLit was translated, culturally adapted, and psychometrically evaluated within the same study sample, and subsequently used to examine its associations with other scales. The overall study procedure is illustrated in Figure 1.

#### 2.2.3 Intuitive eating scale-2 (IES-2)

The IES-2, originally developed by Tylka and Kroon Van Diest (9), assesses individuals' tendency to eat based on internal hunger and satiety cues rather than external or emotional triggers. The Turkish version validated by Bakiner (17) was used in this study. The scale consists of 21 items rated on a 5-point Likert scale (1 = Strongly disagree to 5 = Strongly agree) across four subscales: Unconditional Permission to Eat (items 1–5), Eating for Physical Rather Than Emotional Reasons (items 6–13), Reliance on Hunger and Satiety Cues (items 14–19), and Body-Food Choice Congruence (items 20–21). Items 1, 2, 3, 6, 7, 8, and 9 are reverse-scored. Following the original Turkish validation study, total scores were calculated using only three subscales (excluding Unconditional Permission to Eat) due to its weak loading on the higher-order intuitive eating factor. Higher scores indicate greater intuitive eating. The Turkish version demonstrated good reliability (Cronbach's α = 0.70–0.92 across subscales).

#### 2.2.4 Body appreciation scale (BAS)

The BAS, developed by Avalos, Tylka, and Wood-Barcalow (10), measures individuals' acceptance of, favorable opinions toward, and respect for their bodies. The Turkish version validated by Bakalim and Taşdelen-Karçkay (18) was used. The scale comprises 9 items rated on a 5-point Likert scale (1 = Never to 5 = Always) and consists of two factors: General Body Appreciation (items 1, 2, 3, 4, 5, 8, 9) and Investment in Body Image (items 6, 7). Higher scores indicate greater body appreciation. The Turkish version demonstrated good psychometric properties (Cronbach's α = 0.89–0.92; CFI = 0.93, RMSEA = 0.11).

### 2.3 Linguistic and cultural adaptation

The Turkish adaptation of the S-NutLit followed established guidelines for cross-cultural scale adaptation (19). The process comprised three stages: forward-backward translation, expert panel review, and pilot testing (Figure 1, Phase 1).

The original English version of the S-NutLit was independently translated into Turkish by two bilingual translators. The translations were synthesized into a single version, which was then back-translated into English by two independent translators who were blind to the original version. Discrepancies between the original and back-translated versions were resolved through discussion among the research team. The translated scale was evaluated by a panel of experts in two stages. First, 8 experts assessed translation adequacy using a 4-point scale (1 = Not appropriate to 4 = Fully appropriate). Inter-rater agreement was calculated using Fleiss' Kappa, yielding a value of κ = 0.47 (*p* < 0.001), indicating moderate agreement (20). Overall, 95.5% of ratings were positive (“Appropriate” or “Fully appropriate”).

One item (Item 4, referring to sustainable nutrition) was revised based on expert feedback. Second, 17 experts evaluated content validity using a 5-point comprehensibility scale (1 = Not understandable to 5 = Fully understandable). Content Validity Ratios (CVR) were calculated using Lawshe's method (21), with values ranging from 0.65 to 1.00 across items. The Content Validity Index (CVI), computed as the mean of all CVR values, was 0.93, exceeding the recommended threshold of 0.78 (22).

A pilot study was conducted with 22 young adults from the target population to assess item comprehensibility and response patterns. Item-total correlations ranged from 0.54 to 0.96, indicating adequate item discrimination. No further revisions were required, and the final Turkish version (S-NutLit-TR) was approved for the main study.

### 2.4 Statistical analysis

All statistical analyses were performed using R software (version 4.4.2). The analytical framework is summarized in Figure 1. Data cleaning and transformation were conducted using the dplyr and stringi packages (23, 24). Descriptive statistics and item analyses were computed using the psych package (25). The suitability of data for factor analysis was assessed using the Kaiser-Meyer-Olkin (KMO) measure of sampling adequacy and Bartlett's test of sphericity. Dimensionality was assessed using EGA with the EGAnet package (26). Network estimation employed the graphical LASSO (GLASSO) method, and community detection used the Walktrap algorithm. Structural stability was evaluated through 1,000-iteration parametric bootstrap analysis, examining item replication rates and structural consistency indices. The factor structure identified by EGA was confirmed using CFA with the lavaan package (27). Given the ordinal nature of scale items, the Weighted Least Squares Mean and Variance Adjusted (WLSMV) estimator was used. Four competing models were tested: one-factor, two-factor oblique, two-factor orthogonal, and bifactor models. Model fit was evaluated using χ^2^, Comparative Fit Index (CFI > 0.95), Tucker-Lewis Index (TLI > 0.95), Root Mean Square Error of Approximation (RMSEA < 0.08), and Standardized Root Mean Square Residual (SRMR < 0.08). To address the limitation of conducting EFA and CFA on the same sample, propensity score matching (PSM) was employed using the MatchIt package (28) to create two sociodemographically balanced subsamples (*n* = 170 each). Nearest neighbor matching with a caliper of 0.25 standard deviations was applied across 10 covariates (age, sex, height, weight, education, income, chronic disease, medical nutrition therapy, nutrition education, and physical activity). Target-rotated EFA and Schmid-Leiman (29) bifactor decomposition were conducted on the EFA subsample using the GPArotation package (30) and psych package (25), respectively. The bifactor model was then cross-validated on the independent CFA subsample. Measurement invariance across sex and BMI categories was tested using multi-group CFA with progressively restrictive constraints: configural (same factor structure), metric (equal factor loadings), and scalar (equal item thresholds) invariance. Invariance was evaluated using Chen's (31) criteria: ΔCFI ≤ 0.010, ΔRMSEA ≤ 0.015, and ΔSRMR ≤ 0.030 for metric and ≤ 0.010 for scalar invariance. Internal consistency was assessed using Cronbach's alpha, ordinal alpha, and Guttman's Lambda 6 (G6). Bifactor model-based reliability indices were computed using the semTools package (32), including omega total (ω_t_), omega hierarchical (ω_h_), omega group (ω_g_), composite reliability, explained common variance (ECV), and the H index for factor determinacy. Convergent validity was assessed through Average Variance Extracted (AVE > 0.50) and Pearson correlations between S-NutLit scores and theoretically related constructs (BAS and IES-2). Discriminant (known-groups) validity was examined by comparing S-NutLit scores across groups expected to differ in nutrition literacy (e.g., those with vs. without nutrition education, physical activity levels) using independent samples *t*-tests and one-way ANOVA with LSD *post-hoc* tests. The mediating role of intuitive eating in the relationship between nutrition literacy and body appreciation was tested using structural equation modeling with the lavaan package (27). Indirect effects were estimated using bias-corrected bootstrap confidence intervals (5,000 samples). Separate models were tested for the General factor and Expert Skills factor of S-NutLit as predictors. Figures were created using the ggplot2 and patchwork packages (33, 34). Statistical significance was set at α = 0.05 for all analyses.

### 2.5 Ethical considerations

This study was approved by the Ethics Committee of Istanbul Okan University (Date: 05.06.2024, Decision No: 179). All participants provided informed consent prior to participation. The study was conducted in accordance with the Declaration of Helsinki.

## 3 Results

The final analytical sample comprised 367 young adults (aged 18–24 years). Participant characteristics are presented in Table 1.

**Table 1:** Characteristics of the participants.

Variable	Category	*n*	%
**Sex**	Female	253	68.9
	Male	114	31.1
**Educational level**	Below undergraduate	99	27.0
	Undergraduate or higher	268	73.0
**Income status**	Income lower than expenses	92	25.1
	Income equal to expenses	198	54.0
	Income higher than expenses	77	21.0
**Chronic disease**	Yes	33	9.0
	No	334	91.0
**Medical nutrition therapy**	Yes	17	4.6
	No	350	95.4
**Number of daily meals**	1	16	4.4
	2	191	52.0
	3	136	37.1
	≥4	24	6.5
**Nutrition education**	Yes	138	37.6
	No	229	62.4
**Physical activity level**	None	77	21.0
	Light	171	46.6
	Moderate	77	21.0
	Vigorous	42	11.4
**BMI classification**	Underweight	38	10.4
	Normal weight	231	62.9
	Overweight	72	19.6
	Obese	25	6.8
	NA	1	0.3
Total		367	100.0
**Variable**	***n***	**Range**	**Mean**
**Age (years)**	367	18–24	20.92
**Height (cm)**	367	150–193	168.84
**Weight (kg)**	366	40–172	66.69
**BMI (kg/m** ^**2**^ **)**	366	15.62–58.82	23.21

NA, Participants excluded from BMI classification due to missing height or weight data.

The study sample consisted predominantly of females (68.9%), with males representing 31.1% of participants. Most participants had an undergraduate or higher level of education (73.0%), while 27.0% reported education below the undergraduate level. Regarding income status, more than half of the sample (54.0%) reported that their income was equal to their expenses, whereas 25.1% reported lower income than expenses and 21.0% reported higher income than expenses. A relatively small proportion of participants reported having a chronic disease (9.0%), and only 4.6% indicated that they were receiving medical nutrition therapy. In terms of eating patterns, the majority consumed two meals per day (52.0%), followed by three meals per day (37.1%). Less frequent meal consumption (one meal per day) and higher-frequency consumption (four or more meals per day) were reported by 4.4% and 6.5% of participants, respectively. Nutrition education had been received by 37.6% of the participants, whereas 62.4% reported no prior nutrition education. Physical activity levels were most commonly reported as light (46.6%), followed by none (21.0%), moderate (21.0%), and vigorous activity (11.4%). According to WHO BMI classification, the majority of participants were classified as having normal weight (62.9%), followed by overweight (19.6%), underweight (10.4%), and obese (6.8%). One participant (0.3%) could not be classified due to missing anthropometric data. For continuous variables, the mean age of participants was 20.92 ± 2.05 years, with an age range of 18–24 years. The mean height was 168.84 ± 9.16 cm, the mean body weight was 66.69 ± 17.37 kg, and the mean BMI was 23.21 ± 4.77 kg/m^2^ (Table 1).

### 3.1 Structural validity

Item-level analyses of the original 11-item S-NutLit scale indicated that SNUTLIT7 (Knowing the Türkiye Dietary Guidelines [in Turkish Language: “TÜBER—*Türkiye Beslenme Rehberi*)”] exhibited the lowest corrected item–total correlation (*r* = 0.32), substantially lower than those observed for the remaining items (range: 0.44–0.71). This finding suggested a weaker association of SNUTLIT7 with the overall nutrition literacy construct. Comprehensive item-level descriptive statistics, including means, standard deviations, skewness, kurtosis, and response distributions, are reported in Supplementary Table S1.

To examine the factor structure of the S-NutLit scale, Exploratory Graph Analysis (EGA) was employed as a contemporary network psychometric approach for dimensionality assessment. Developed by Golino and Epskamp (35), EGA estimates a network structure based on partial correlations among variables and applies community detection algorithms to determine the number of dimensions. Unlike traditional factor analysis, EGA demonstrates lower sensitivity to sample size, reduced tendency for over-factorization, and provides visually interpretable outputs. Network estimation was performed using the graphical LASSO (GLASSO) method, and community detection utilized the Walktrap algorithm. To comprehensively evaluate structural validity, parametric bootstrap analysis with 1,000 iterations was conducted to compute item and dimension stability statistics. Detailed item-level descriptive statistics, including means, standard deviations, skewness, kurtosis, corrected item–total correlations, and response distributions, are provided in Supplementary Table S1.

**Figure 2:**
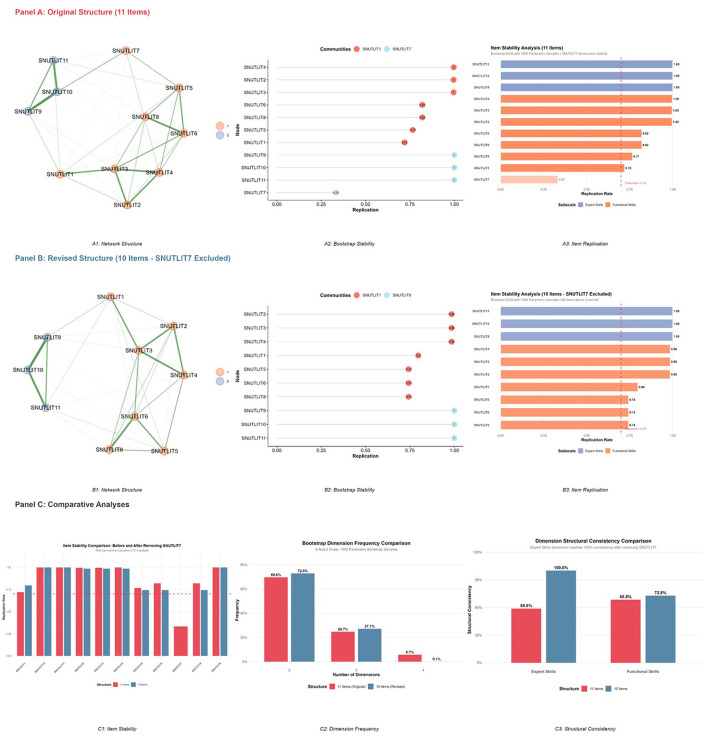
Exploratory graph analysis (EGA) network structure of the final 10-item S-NutLit scale. Panel **(A)** presents the original 11-item structure: **(A1)** network plot with GLASSO estimation and Walktrap community detection showing two communities (Information Skills in coral/salmon; Expert Skills in teal) with SNUTLIT7 displaying weak connections; **(A2)** bootstrap stability analysis (1,000 iterations) showing item replication rates by dimension; **(A3)** detailed item replication rates with SNUTLIT7 exhibiting critically low stability (33.3%). Panel **(B)** presents the revised 10-item structure following SNUTLIT7 removal: **(B1)** refined network plot with clearer community separation; **(B2)** improved bootstrap stability with all items above the 70% threshold; **(B3)** enhanced item replication rates ranging from 79.7% to 100%. Panel **(C)** displays comparative analyses: **(C1)** item stability comparison before (blue) and after (red) SNUTLIT7 removal; **(C2)** bootstrap dimension frequency showing increased two-factor solutions (69.6% → 72.8%) and dramatic reduction in four-factor solutions (5.7% → 0.1%); **(C3)** structural consistency comparison demonstrating Expert Skills improvement from 58.8% to 100% following item removal. Edge thickness in network plots reflects partial correlation strength; node colors indicate community membership.

EGA conducted on the original 11-item S-NutLit scale revealed a two-dimensional structure. The first dimension comprised eight items measuring access to and evaluation of nutrition information (SNUTLIT1–SNUTLIT8; Information Skills), while the second dimension included three items measuring consultation with expert sources and seeking professional guidance (SNUTLIT9–SNUTLIT11; Expert Skills). This two-factor structure aligned fully with the original scale structure developed by Vrinten et al. (7). In the estimated network structure, strong positive partial correlations were observed among items within the same dimension, whereas connections between items from different dimensions were relatively weak. Notably, the three Expert Skills items formed a dense cluster clearly differentiated from the first dimension. In the network visualization, SNUTLIT3 was positioned at the center of the Information Skills dimension, while SNUTLIT10 constituted the focal point of the Expert Skills dimension (Figure 2).

To evaluate the stability of the two-dimensional structure, parametric bootstrap analysis with 1000 replications was performed. Bootstrap results indicated that a two-dimensional structure emerged in 69.6% of samples, a three-dimensional structure in 24.7%, and a four-dimensional structure in 5.7%. The median number of dimensions was 2, with a 95% confidence interval ranging from 0.85 to 3.15. These findings suggest that while the two-dimensional structure was predominant, alternative structures emerged in approximately one-third of samples, indicating structural uncertainty for certain items. This variation in dimension number reflected inconsistent item assignments across bootstrap samples. Item-level stability analyses provided further insight into this pattern.

Item stability analysis revealed replication rates indicating how frequently each item was assigned to the same dimension across bootstrap samples. Using the 70% threshold recommended by Christensen and Golino (36), the majority of items demonstrated acceptable stability. The three Expert Skills items (SNUTLIT9, SNUTLIT10, SNUTLIT11) exhibited perfect stability with 100% replication rates, consistently assigned to the same dimension across all bootstrap samples. Within the Information Skills dimension, SNUTLIT2 (99.6%), SNUTLIT3 (99.6%), and SNUTLIT4 (99.7%) showed very high stability, while SNUTLIT5 (76.6%), SNUTLIT6 (82.0%), and SNUTLIT8 (82.0%) demonstrated moderate-to-high stability. SNUTLIT1 (72.0%) was positioned just above the threshold. However, SNUTLIT7 exhibited a critically low replication rate of only 33.3%, indicating serious structural problems. Examination of SNUTLIT7's distribution across bootstrap samples revealed assignment to the first dimension in 23.4% of cases, to the second dimension in 33.3%, to a third dimension in 12.3%, and to a fourth dimension in 5.5%. This distribution demonstrates that SNUTLIT7 failed to show clear dimensional affiliation and exhibited a near-random assignment pattern.

Dimension stability analysis computed structural consistency values indicating how frequently each dimension was replicated with exactly the same items across bootstrap samples. In the 11-item structure, structural consistency was 0.685 for Information Skills and 0.588 for Expert Skills. The lower consistency of the Expert Skills dimension was attributable to SNUTLIT7 occasionally being assigned to this dimension. Mean item stability values were 87.4% for Information Skills and 83.3% for Expert Skills.

Network loadings, conceptually analogous to factor loadings, indicate the strength of each item's relationship with its dimension. This metric, developed by Christensen and Golino (36), reflects the sum of partial correlations between an item and other items within its dimension. In the 11-item structure, SNUTLIT3 showed the highest loading on the Information Skills dimension (0.678), followed by SNUTLIT6 (0.564), SNUTLIT4 (0.546), SNUTLIT2 (0.493), SNUTLIT5 (0.443), SNUTLIT8 (0.419), and SNUTLIT1 (0.239). SNUTLIT7 showed a loading of only 0.103 on the first dimension, nearly equivalent to its loading on the second dimension (0.095). These low and ambiguous loadings indicate that the item established weak relationships with both dimensions and made no functional contribution to the scale. The Expert Skills dimension showed strong loadings for SNUTLIT10 (0.647), SNUTLIT9 (0.536), and SNUTLIT11 (0.465), with minimal cross-loadings.

Weighted Topological Overlap (wTO) analysis was conducted to assess local dependence among item pairs. This analysis reveals the degree of redundancy between item pairs and identifies items potentially measuring the same content. Using the 0.25 threshold recommended by Christensen, Garrido, and Golino (37), high overlap was observed among Expert Skills items: SNUTLIT9–SNUTLIT10 (wTO = 0.379), SNUTLIT10–SNUTLIT11 (0.329), and SNUTLIT9–SNUTLIT11 (0.251), indicating large redundancy. These findings suggest that the three Expert Skills items measure conceptually very similar content. Within the Information Skills dimension, moderate redundancy was observed for SNUTLIT6–SNUTLIT8 (0.281), SNUTLIT2–SNUTLIT3 (0.278), and SNUTLIT3–SNUTLIT4 (0.262). These local dependence patterns, while providing high internal consistency, also suggest that certain items share similar variance. Complete bootstrap stability and local dependence results are presented in Supplementary Tables S2, S3.

Bootstrap analysis on the 10-item structure revealed marked improvements in structural stability. The frequency of two-dimensional solutions increased from 69.6% to 72.8%, while four-dimensional solutions dramatically decreased from 5.7% to 0.1%. This dramatic reduction clearly demonstrates the extent to which SNUTLIT7 contributed to structural noise. The 95% confidence interval for the median number of dimensions narrowed from [0.85, 3.15] to [1.12, 2.88], reflecting reduced uncertainty in dimension estimation. All items maintained replication rates above the 70% threshold, with SNUTLIT1's stability improving from 72.0% to 79.7%. Most notably, the structural consistency of the Expert Skills dimension increased from 58.8% to 100%, meaning that following SNUTLIT7's removal, the Expert Skills dimension was replicated with exactly the same three items in every bootstrap sample. The Information Skills dimension's structural consistency also improved from 68.5% to 72.8%.

In the 10-item structure, standardized network loadings exhibited a cleaner pattern. Information Skills loadings were: SNUTLIT3 (0.687), SNUTLIT6 (0.565), SNUTLIT4 (0.509), SNUTLIT2 (0.492), SNUTLIT8 (0.415), SNUTLIT5 (0.374), and SNUTLIT1 (0.249). Expert Skills loadings were: SNUTLIT10 (0.653), SNUTLIT9 (0.536), and SNUTLIT11 (0.468). All cross-loadings remained below 0.10, indicating a clean factor structure. Reliability analyses also supported the psychometric strength of the 10-item structure, with Cronbach's alpha increasing from 0.87 to 0.88 for the total scale and from 0.88 to 0.90 for the Information Skills subscale, while Expert Skills remained stable at 0.82.

Evaluating EGA findings as a whole, the two-dimensional structure of the Turkish S-NutLit was generally supported, although SNUTLIT7 did not function as expected in the Turkish sample. This item's low item–total correlation (*r* = 0.32), low bootstrap stability (33.3%), low network loading (0.103), and ambiguous dimensional affiliation were notable findings. SNUTLIT7 originally contained the statement “I know the basic rules of the Flemish Food Triangle” and was culturally adapted in the Turkish version as “I know the basic rules of the Türkiye Dietary Guidelines (TÜBER).” Unlike other Information Skills items, this item measures knowledge of a specific national dietary guideline. Following SNUTLIT7's removal, substantial improvements were observed in structural consistency, item stability, and reliability: Expert Skills structural consistency increased from 58.8% to 100%, two-dimensional structure frequency in bootstrap samples increased from 69.6% to 72.8%, and Information Skills Cronbach's alpha increased from 0.88 to 0.90. Consequently, the decision was made to exclude SNUTLIT7.

EGA, as a data-driven exploratory approach, determined the number of dimensions and item-dimension relationships; however, confirmatory factor analysis (CFA) was required to statistically validate the identified structure. Unlike EGA, CFA adopts a hypothesis-driven approach, testing the extent to which a predetermined factor structure fits the observed data. Given the ordinal nature of scale items, the Weighted Least Squares Mean and Variance Adjusted (WLSMV) estimator was employed.

Four models were tested within the CFA framework: one-factor, two-factor oblique (correlated factors), two-factor orthogonal (uncorrelated factors), and bifactor. No error covariances were specified in any model, and no modifications were required as model results fell within recommended index ranges. Complete model comparison results are presented in Supplementary Table S4.

The one-factor model showed poor fit to the data. Although the CFI value (0.956) approached the acceptable threshold, RMSEA (0.229) and SRMR (0.148) substantially exceeded acceptable limits, indicating that the 10 items cannot be adequately represented by a single general nutrition literacy factor. The two-factor orthogonal model, assuming no correlation between factors, showed even poorer fit than the one-factor model, demonstrating that the Information Skills and Expert Skills factors are not independent and share meaningful common variance. The two-factor oblique model showed excellent fit (CFI = 0.996, TLI = 0.995, RMSEA = 0.069, SRMR = 0.050).

The bifactor model achieved the best fit indices, with a non-significant chi-square test (*p* = 0.156) indicating excellent model–data correspondence. In the bifactor model, all items load simultaneously on a general factor and their respective specific factors. Examination of bifactor loadings revealed a critical pattern: Information Skills items (SNUTLIT1–6, SNUTLIT8) showed high and significant loadings on the general factor (0.655–0.915) but low loadings on the Information-specific factor (−0.303 to 0.144), most of which were statistically non-significant. In contrast, Expert Skills items (SNUTLIT9–11) loaded significantly on both the general factor (0.295–0.409) and the Expert-specific factor (0.651–0.846), with specific factor loadings substantially higher than general factor loadings.

This pattern indicates that the variance of Information Skills items is largely explained by the general factor, and these items do not constitute a separate specific dimension—in other words, Information Skills items already represent the general nutrition literacy construct. Expert Skills items, however, contribute both to the general construct and explain unique variance beyond the general factor. This is theoretically meaningful: Expert Skills encompass advanced competencies such as evaluating nutrition information sources, distinguishing nutrition experts from non-experts, and critically reading nutrition news—skills that build upon yet remain distinct from basic nutrition literacy. Accordingly, the final bifactor model excluded the Information-specific factor and retained only the General Factor and Expert-specific factor (Supplementary Figure S1).

The final bifactor model demonstrated excellent fit (CFI = 0.997, TLI = 0.996, RMSEA = 0.062, SRMR = 0.043). All items loaded significantly on the general factor, with Information Skills items showing strong loadings (0.650–0.910) and Expert Skills items showing moderate general factor loadings (0.295–0.408) alongside strong Expert-specific loadings (0.651–0.847). Communality values ranged from 0.423 to 0.828, with the highest values observed for SNUTLIT3 (0.828) and SNUTLIT10 (0.804). Standardized factor loadings, communalities, and reliability indices are presented in Table 2.

**Table 2:** Factor loadings and reliability indices of the final bifactor model of the S-NutLit scale (*n* = 367).

Item	Item content	λ_General_	λ_Expert_	h^2^
SNUTLIT1	Understanding food labels	0.650^***^	—	0.423
SNUTLIT2	Comparing nutritional values	0.845^***^	—	0.714
SNUTLIT3	Using portion information	0.910^***^	—	0.828
SNUTLIT4	Understanding daily reference values	0.855^***^	—	0.731
SNUTLIT5	Calculating calories	0.728^***^	—	0.530
SNUTLIT6	Making healthy choices	0.845^***^	—	0.714
SNUTLIT8	Evaluating food content	0.770^***^	—	0.593
SNUTLIT9	Evaluating nutrition experts	0.408^***^	0.697^***^	0.653
SNUTLIT10	Evaluating nutrition information sources	0.295^***^	0.847^***^	0.804
SNUTLIT11	Critically reading nutrition news	0.380^***^	0.651^***^	0.568
Index	Threshold	General	Expert	Interpretation
**Cronbach** **α**	>0.70	0.880	0.816	High internal consistency
**Ordinal** **α**	>0.70	0.900	0.854	High internal consistency accounting for Likert scaling
**Omega (ω)**	>0.70	0.771	0.692	Higher reliability for the general factor
**AVE**	>0.50	0.647	0.542	Both subscale scores can be confidently reported
**ECV**		0.752	0.248	Dominant variance explained by the general factor
**H index**	>0.80	0.942	0.808	Both factors are well defined
**ω_h_**	>0.30	0.493	—	Supports reporting both total and subscale scores

λ_General_, general factor loading; λ_Expert_, expert-specific factor loading; h^2^, communality; AVE, Average Variance Extracted; ECV, Explained Common Variance; ω_h_, omega hierarchical; Model fit indices, χ^2^_(32)_ = 77.39, *p* < 0.001; CFI: 0.997; TLI: 0.996; RMSEA: 0.062 [0.045, 0.080]; SRMR: 0.043. ^***^p < 0.001.

Reliability indices exceeded acceptable levels for both factors. Cronbach's alpha values were .880 for the General Factor and 0.816 for Expert Skills. Ordinal alpha values (0.900 and 0.854) were higher than standard Cronbach's alpha, indicating that accounting for the categorical structure of ordinal data provides more accurate reliability estimates. Composite reliability (omega) values were 0.771 for the General Factor and 0.692 for Expert Skills; the Expert factor falling slightly below the 0.70 threshold is attributable to its three-item structure and remains acceptable. Average Variance Extracted (AVE) values exceeded the 0.50 threshold for both factors (General = 0.647; Expert = 0.542), supporting convergent validity. Bifactor-specific indices provided important information about the model structure. The ECV value (0.752) indicated that the general factor explained 75.2% of the common variance, signifying its dominance in the scale. H indices exceeded 0.80 for both the General Factor (0.942) and Expert-specific factor (0.808), indicating well-defined constructs replicable across samples. The omega hierarchical (ω_h_) value of 0.493, while above the 0.30 threshold supporting total score use, fell below 0.70, indicating the importance of also reporting Expert Skills subscale scores (Table 2).

Based on these findings, the Turkish S-NutLit is recommended for use with both total scores and Expert Skills subscale scores. The General Factor represents a broad construct encompassing fundamental nutrition literacy skills (label reading, comparing nutritional values, using portion information, etc.). The Expert Skills specific factor reflects advanced competencies beyond these fundamental skills, including critical evaluation of nutrition information sources and distinguishing experts from pseudo-experts. This two-level structure offers the opportunity to separately measure both the general level of nutrition literacy and the critical evaluation dimension.

### 3.2 Sensitivity analysis

A gold standard in psychometric research is the use of separate samples for exploratory and confirmatory analyses, as conducting both on the same dataset creates circular logic—EFA derives structure from data, while CFA tests the fit of a predetermined structure (15). When both analyses are conducted on the same sample, the CFA “confirms” a structure already derived from that data, meaning the confirmation is no longer a truly independent test. However, for scales with fewer than 20 items, a minimum of 200 participants is recommended for factor analysis. When total sample size does not reach 400, as in the present study, conducting exploratory and confirmatory analyses on the same sample becomes unavoidable. To address this limitation, propensity score matching (PSM) was employed to create two demographically balanced subsamples. While random splitting is an option, simple random division can create systematic differences between groups (e.g., higher mean age in one group, higher proportion of females in another). In such cases, it becomes unclear whether different EFA and CFA results stem from structural differences or sample characteristics. PSM solves precisely this problem by matching individuals based on sociodemographic characteristics, ensuring EFA and CFA groups are as similar as possible.

PSM was conducted using 10 sociodemographic covariates identified as factors potentially influencing nutrition literacy: age, sex, height, weight, education level, income status, chronic disease, medical nutrition therapy, nutrition education, and physical activity level. Nearest-neighbor matching with a 1:1 ratio and 0.25 SD caliper was employed, with propensity scores estimated via generalized linear model (GLM). Following matching, 170 participants were assigned to each subsample, with 26 participants remaining unmatched. All standardized mean differences were below .10 (age: 0.011; height: −0.051; weight: −0.061), indicating virtually identical groups on these variables. Statistical tests confirmed group equivalence: *t*-tests for continuous variables (age: t = −0.11, *p* = 0.916; height: *t* = 0.47, *p* = 0.638; weight: *t* = 0.56, *p* = 0.573) and chi-square tests for categorical variables (all *p* > 0.05) demonstrated statistical equivalence. Complete matching diagnostics are presented in Supplementary Figure S2 and Supplementary Table S5.

Target-rotated EFA with Schmid–Leiman decomposition was conducted in the EFA subsample (*n* = 170). Prior to analysis, KMO (0.889) and Bartlett's test (χ^2^ = 1,113.10, *p* < 0.001) confirmed data suitability. The target matrix was constructed based on bifactor model findings. Results largely supported the predetermined structure: all items loaded strongly on the general factor (0.772–1.006), while only Expert Skills items showed meaningful loadings on the Expert factor (0.895–1.134). The Schmid–Leiman solution revealed moderate and homogeneous general factor loadings (0.46–0.58). Model fit was acceptable (RMSEA = 0.092, TLI = 0.938), with two factors explaining 65.3% of total variance. Omega total (0.93) indicated high overall reliability, while omega hierarchical (0.51) suggested approximately half of reliable variance was attributable to the general factor. Complete EFA results are presented in Supplementary Table S6.

Cross-validation CFA in the independent CFA subsample (*n* = 170) demonstrated good model fit (CFI = 0.993, TLI = 0.990, RMSEA = 0.077, SRMR = 0.065). Factor loadings closely replicated those from the full sample: Information Skills items loaded 0.575–0.911 on the general factor, while Expert Skills items showed the expected bifactor pattern (general: 0.181–0.394; Expert-specific: 0.602–0.874). Bifactor indices were consistent with full-sample results (ECV = 0.731 vs. 0.752 in full sample; H indices > 0.80 for both factors; ωh = 0.429), providing robust support for the stability and replicability of the proposed bifactor structure. Complete cross-validation results are presented in Supplementary Table S7.

### 3.3 Measurement invariance

Measurement invariance of the bifactor model was tested across sex (male vs. female) and BMI classification (normal/underweight vs. overweight/obese) using the full sample (*N* = 367). Three levels were tested hierarchically: configural, metric, and scalar invariance. Since variances are fixed for categorical variables with the WLSMV estimator, strict invariance was not applicable in this context. Results are presented in Table 3.

**Table 3:** Measurement invariance results for the S-NutLit Bifactor model across sex and BMI classification.

Grouping variable	Fit indices	Configural^1^	Metric^2^	Scalar^3^
Sex	CFI	0.998	0.996 (Δ_2 − 1_ = −0.002)	0.999 (Δ_3 − 2_ = +0.003)
	TLI	0.998	0.996 (Δ_2 − 1_ = −0.002)	0.999 (Δ_3 − 2_ = +0.003)
	RMSEA	0.051	0.066 (Δ_2 − 1_ = +0.015)	0.032 (Δ_3 − 2_ = −0.034)
	SRMR	0.047	0.054 (Δ_2 − 1_ = +0.007)	0.049 (Δ_3 − 2_ = −0.005)
BMI Classification	CFI	0.997	0.997 (Δ_2 − 1_ = −0.000)	0.999 (Δ_3 − 2_ = −0.028)
	TLI	0.996	0.996 (Δ_2 − 1_ = +0.000)	0.999 (Δ_3 − 2_ = +0.003)
	RMSEA	0.066	0.064 (Δ_2 − 1_ = −0.002)	0.036 (Δ_3 − 2_ = −0.028)
	SRMR	0.051	0.055 (Δ_2 − 1_ = +0.004)	0.052 (Δ_3 − 2_ = −0.003)

*N* = 367. CFI, Comparative Fit Index; TLI, Tucker–Lewis Index; RMSEA, Root Mean Square Error of Approximation; SRMR, Standardized Root Mean Square Residual. Δ indicates change relative to the previous invariance level. BMI classification groups were defined as normal or below (underweight + normal weight) vs. overweight or above (overweight + obese). ^1^Configural invariance; ^2^Metric invariance; ^3^Scalar invariance. Measurement invariance was evaluated using the criteria proposed by Chen (31): for metric invariance, ΔCFI ≤ 0.010, ΔRMSEA ≤ 0.015, ΔSRMR ≤ 0.030; for scalar (and strict) invariance, ΔCFI ≤ 0.010, ΔRMSEA ≤ 0.015, ΔSRMR ≤ 0.010.

For sex, the configural model showed excellent fit (CFI = 0.998, TLI = 0.998, RMSEA = 0.051), confirming the validity of the bifactor structure in both male and female groups. The transition to metric invariance resulted in changes within acceptable limits (ΔCFI = −0.002, ΔSRMR = +0.007), with ΔRMSEA (+0.015) at the exact boundary value of Chen's (31) criteria. Scalar invariance showed improved fit (ΔCFI = +0.003, ΔRMSEA = −0.034), with all criteria met (Table 3).

For BMI classification, a similar pattern emerged: the configural model showed good fit (CFI = 0.997, TLI = 0.996, RMSEA = 0.066), metric invariance was comfortably achieved (ΔCFI = 0.000, ΔRMSEA = −0.002), and scalar invariance showed marked improvement (ΔCFI = +0.002, ΔRMSEA = −0.028) (Table 3).

The establishment of scalar invariance for both variables indicates that the Turkish S-NutLit measures the same construct equivalently across sex and BMI groups, with equivalent factor loadings and item threshold values. Consequently, mean comparisons between groups are meaningful and reliable, with observed score differences reflecting true structural differences rather than measurement error.

### 3.4 Reliability and validity evidence

Convergent validity was examined through correlations with theoretically related constructs. The S-NutLit General factor showed a strong positive correlation with the Expert factor (*r* = 0.673, *p* < 0.001), confirming that the two factors measure related but distinct aspects of the same overarching construct. The General factor demonstrated significant positive correlations with the Intuitive Eating Scale-2 (*r* = 0.416, *p* < 0.001) and Body Appreciation Scale (*r* = 0.236, *p* < 0.001). The Expert factor showed weaker but significant correlations with IES-2 (*r* = 0.186, *p* < 0.001) and BAS (*r* = 0.114, *p* = 0.029). The stronger correlation between the General factor and IES-2 indicates that general nutrition literacy is closely related to intuitive eating behaviors, while the Expert factor's lower correlations suggest it captures a unique and distinct skill set. Complete correlation results are presented in Supplementary Table S8.

Known-groups validity analyses revealed that the S-NutLit successfully discriminates between groups expected to differ in nutrition literacy. For the General factor, significantly higher scores were observed among individuals with nutrition education (*t* = 5.23, *p* < 0.001) and medical nutrition therapy experience (*t* = 3.47, *p* < 0.001). Participants consuming 2–3 meals daily showed higher scores than those consuming only one meal (*F* = 2.77, *p* = 0.041). For the Expert factor, females scored significantly higher than males (*t* = −2.14, *p* = 0.033), and significant differences emerged for nutrition education (*t* = 6.18, *p* < 0.001), medical nutrition therapy (*t* = 3.82, *p* < 0.001), and physical activity level (*F* = 3.93, *p* = 0.009), with active individuals scoring higher than sedentary individuals. Body Appreciation scores differed by income status (*F* = 6.45, *p* = 0.002) and BMI classification (*F* = 10.06, *p* < 0.001), with higher-income participants and normal/underweight individuals reporting greater body appreciation. No significant differences were observed for education level, chronic disease, or Intuitive Eating scores (*p* > 0.05). These findings demonstrate the scale's ability to differentiate individuals based on nutrition-related experiences and behaviors, supporting discriminant validity. Complete known-groups comparisons are provided in Supplementary Table S9.

### 3.5 Mediation analysis

**Figure 3:**
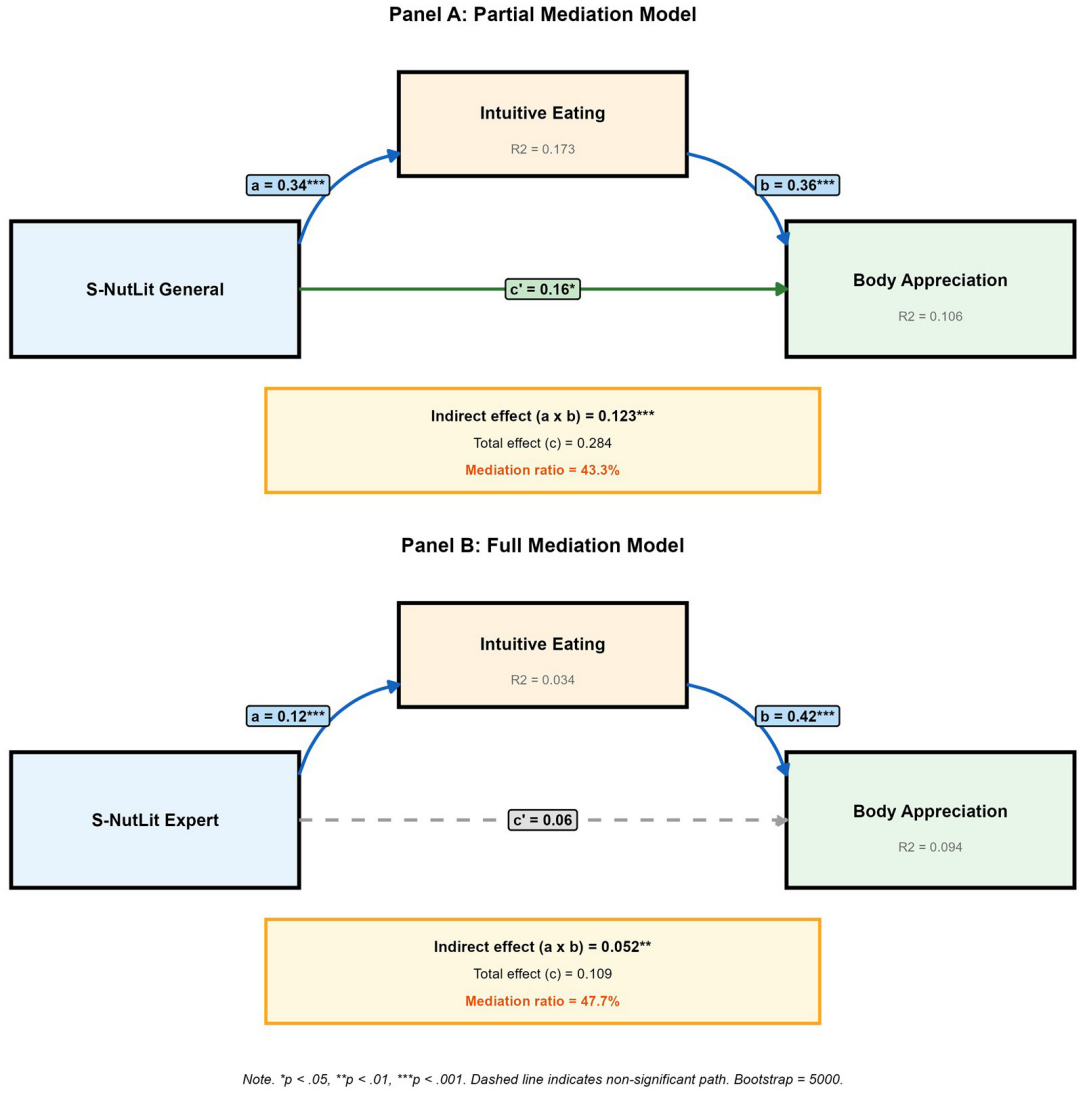
Mediation models examining the associations between nutrition literacy and body appreciation through intuitive eating. Panel **(A)** displays the partial mediation model with S-NutLit General factor as the predictor. Panel **(B)** displays the full mediation model with S-NutLit Expert factor as the predictor. Path coefficients represent unstandardized regression weights. Solid lines indicate statistically significant paths; dashed line indicates a non-significant path. R^2^ values represent the proportion of variance explained in the mediator (Intuitive Eating) and outcome (Body Appreciation) variables. Indirect effects were tested using bias-corrected bootstrap confidence intervals with 5,000 resamples. *p < 0.05, **p < 0.01, ***p < 0.001.

Two mediation models were tested to examine the role of intuitive eating in the relationship between nutrition literacy and body appreciation. In Model 1, the S-NutLit General factor was the independent variable; in Model 2, the Expert factor was the independent variable. In both models, intuitive eating served as the mediator and body appreciation as the dependent variable. Indirect effect significance was tested using 5,000 bootstrap samples. Results are presented in Table 4 and Figure 3.

**Table 4:** The mediating role of intuitive eating in the association between nutrition literacy and body appreciation (*n* = 367).

Model	Path	B	SE	β	%95 CI	*p*
*Model 1*: S*-NutLit General*						
	a (X → M)	0.342	0.05	0.416	[0.238, 0.441]	< 0.001^***^
	b (M → Y)	0.358	0.08	0.246	[0.195, 0.525]	< 0.001^***^
	c' (X → Y, direct)	0.161	0.07	0.134	[0.021, 0.299]	0.022^*^
	a × b (indirect)	0.123	0.03	0.102	[0.062, 0.197]	< 0.001^***^
	c (total)	0.284	0.07	0.236	[0.148, 0.421]	< 0.001^***^
	R^2^_(M)_ = 0.173; R^2^_(Y)_ = 0.106					
*Model 2*: S*-NutLit Expert*						
	a (X → M)	0.122	0.03	0.186	[0.049, 0.193]	< 0.001^***^
	b (M → Y)	0.423	0.08	0.290	[0.261, 0.591]	< 0.001^***^
	c' (X → Y, direct)	0.057	0.05	0.060	[−0.045, 0.153]	0.257
	a × b (indirect)	0.052	0.01	0.054	[0.019, 0.094]	0.007^**^
	c (total)	0.109	0.05	0.114	[0.003, 0.209]	0.038^*^
	R^2^_(M)_ = 0.034; R^2^_(Y)_ = 0.094					

X, Independent variable (S-NutLit); M, Mediator (Intuitive Eating Scale); Y, Dependent variable (Body Appreciation Scale). B, unstandardized regression coefficient; SE, standard error; β, standardized regression coefficient; CI, confidence interval. Bootstrap resampling was performed with 5,000 samples. R^2^(M) indicates the variance explained in the mediator; R^2^(Y) indicates the variance explained in the outcome. ^*^p < 0.05; ^**^p < 0.01; ^***^p < 0.001.

In Model 1, the S-NutLit General factor significantly predicted intuitive eating (a = 0.342, β = 0.416, *p* < 0.001), which in turn significantly predicted body appreciation (b = 0.358, β = 0.246, *p* < 0.001). The indirect effect was statistically significant [ab = 0.123, 95% CI (0.062, 0.197), *p* < 0.001], and the direct effect remained significant (c' = 0.161, β = 0.134, *p* = 0.022), indicating partial mediation. The indirect effect accounted for 43.3% of the total effect. Model 1 explained 17.3% of intuitive eating variance and 10.6% of body appreciation variance (Table 4). The mediation model can be expressed as follows:


$$\begin{array}{r} Intuitive\;Eating=\beta_{0}+0.342\\ \times \;SNutLit_{General}+\varepsilon_{1},\\ Body\;Appreciation=\beta_{0}+0.161\times \;SNutLit_{General}+0.358\\ \;\times Intuitive\;Eating+\varepsilon_{2}. \end{array}$$

For Model 1, the indirect effect of S-NutLit (General) on Body Appreciation through Intuitive Eating was calculated as *a*×*b* = 0.342 × 0.358 = 0.123.

In Model 2, the S-NutLit Expert factor significantly predicted intuitive eating (a = 0.122, β = 0.186, *p* < 0.001), which predicted body appreciation (b = 0.423, β = 0.290, *p* < 0.001). The indirect effect was significant [ab = 0.052, 95% CI (0.019, 0.094), *p* = 0.007]. However, the direct effect was non-significant (c' = 0.057, β = 0.060, *p* = 0.257), indicating full mediation. The indirect effect accounted for 47.7% of the total effect (c = 0.109). Model 2 explained 3.4% of intuitive eating variance and 9.4% of body appreciation variance (Table 4). The mediation model for Model 2 can be specified as:


$$\begin{array}{r} Intuitive\;Eating=\beta_{0}+0.122\\ \times \;SNutLit_{Expert}+\varepsilon_{1}\\ Body\;Appreciation=\beta_{0}+0.057\times \;SNutLit_{Expert}+0.423\\ \times Intuitive\;Eating+\varepsilon_{2} \end{array}$$

Accordingly, the indirect effect of S-NutLit (Expert) on Body Appreciation via Intuitive Eating was calculated as *a*×*b* = 0.122 × 0.423 = 0.053.

The difference in mediation patterns between the two models provides important insights into the nature of the relationships between the two factors of the S-NutLit scale and body appreciation (Figure 3).

The differential mediation patterns between the two models offer important insights into the nature of each factor's relationship with body appreciation. For general nutrition literacy (partial mediation), the significant direct effect suggests that basic nutrition skills may influence body appreciation through multiple pathways beyond intuitive eating, such as perceived competence in making healthy food choices, reduced anxiety about eating decisions, and sense of control over one's body. For expert-level skills (full mediation), the effect on body appreciation operates entirely through intuitive eating. This suggests that critical nutrition literacy skills were not directly associated with body appreciation. Instead, they may be linked to intuitive eating behaviors by enabling individuals to trust their internal hunger-satiety signals, reduce reliance on external pressures, and develop a healthier relationship with their body. In other words, individuals capable of critically evaluating nutrition information sources question societal messages about diets and body ideals, which facilitates adoption of intuitive eating approaches and ultimately contributes to greater body satisfaction. Both models confirm intuitive eating as an important mediating variable in the nutrition literacy–body appreciation relationship, while the differential mechanisms indicate that different dimensions of nutrition literacy influence body appreciation through distinct pathways. These findings also provide evidence for predictive validity (Figure 3). It is important to emphasize that these findings reflect statistical associations and indirect pathways observed in cross-sectional data rather than causal effects. Although the proposed models are theoretically grounded, the temporal ordering of nutrition literacy, intuitive eating, and body appreciation cannot be empirically established within the present design. Accordingly, the mediation results should be interpreted as evidence of plausible relational mechanisms rather than definitive causal processes.

## 4 Discussion

This study provides the first comprehensive validation of the Turkish adaptation of the Short Nutrition Literacy Scale (S-NutLit-TR) using contemporary psychometric approaches, while simultaneously examining the mediating role of intuitive eating in the relationship between nutrition literacy and body appreciation among Turkish young adults.

The importance of measuring nutrition literacy in young adults is underscored by our finding that participants who had received medical nutrition therapy demonstrated significantly higher general nutrition literacy scores (3.89 vs. 3.54, *p* < 0.01). This 0.35-point difference on a 5-point scale represents a meaningful practical effect, suggesting that professional nutrition counseling was associated with higher nutrition literacy levels. Conversely, participants consuming only one meal per day exhibited significantly lower general nutrition literacy (3.21 vs. 3.58, *p* < 0.05). This finding is particularly concerning given that 4.4% of our sample reported this eating pattern. Meessen et al. (38) demonstrated that while single-meal consumption may yield short-term metabolic benefits in controlled settings, such patterns in naturalistic contexts often reflect disordered eating behaviors rather than intentional intermittent fasting. Our data suggest that young adults with lower nutrition literacy was associated with patterns that may reflect greater susceptibility to adopting potentially harmful dietary practices promoted through social media, mistaking them for evidence-based approaches. The structural differences between our Turkish bifactor model and the original Belgian two-factor oblique structure reported by Vrinten et al. (7) warrant attention. While they found information skills and expert skills as distinct but correlated factors (*r* = 0.67), our analysis revealed that information skills items loaded primarily onto a general factor (λ = 0.52–0.71), whereas expert skills demonstrated dual loading on both general (λ = 0.41–0.58) and specific (λ = 0.34–0.52) factors. This pattern suggests that in the Turkish context, basic nutrition information processing may be more closely integrated with overall nutrition literacy, while critical evaluation skills retain unique variance.

The emergence of the bifactor structure has important theoretical implications for understanding nutrition literacy dimensionality. Our model yielded strong bifactor indices: the explained common variance (ECV = 0.752) indicated that 75.2% of the common variance was attributable to the general factor, while the remaining 24.8% was explained by the expert-specific factor. The Explained Common Variance was above the 0.60 guideline frequently used in the interpretation of bifactor models (39), indicating a dominant general nutrition literacy factor alongside a substantively relevant expert skills dimension. This finding aligns with Nutbeam's (40) hierarchical health literacy model, which posits functional literacy as foundational, interactive literacy as intermediate, and critical literacy as the highest tier. Our expert skills factor—capturing abilities to evaluate nutrition experts (SNUTLIT9), evaluate nutrition information sources (SNUTLIT10), and critically read nutrition news (SNUTLIT11)—conceptually corresponds to critical nutrition literacy. McNamara et al. (2, 41) similarly found through qualitative and quantitative work that critical nutrition literacy, encompassing skills related to evaluating media claims and evidence-based sources, operates as a distinct competency among young adults. Their YA-NLT validation revealed that critical literacy items clustered separately from functional items in both EFA (factor loadings 0.62–0.78) and CFA, paralleling our structural findings.

The mediation analyses revealed differential pathways through which nutrition literacy dimensions are associated with body appreciation through distinct pathways. In Model 1 (general nutrition literacy → intuitive eating → body appreciation), the total effect was significant (β = 0.236, *p* < 0.001), decomposed into a direct effect (β = 0.134, *p* < 0.022) and indirect effect (β = 0.102, *p* < 0.001). The indirect effect accounted for 43.3% of the total effect, indicating partial mediation. However, Model 2 (expert skills → intuitive eating → body appreciation) demonstrated a strikingly different pattern: the total effect (β = 0.114, *p* < 0.038) was entirely mediated through intuitive eating, with a non-significant direct effect (β = 0.060, *p* = 0.257) and significant indirect effect (β = 0.054, *p* < 0.007) accounting for 47.7% of the total effect. This full mediation for expert skills has important theoretical implications: Why might expert-level nutrition skills appear to be associated with body appreciation primarily through intuitive eating? We propose that critical evaluation competencies may be associated with a greater tendency to recognize and question misleading dietary messages, such as “clean eating” rules, detox claims, or restrictive diet trends, that undermine intuitive eating. Individuals who can critically appraise nutrition information may be less likely to internalize food rules that disconnect them from internal hunger and satiety cues. Messer et al. (42) provided longitudinal evidence supporting this mechanism: in their three-wave study (*N* = 3,039 women), body appreciation at Time 1 predicted unconditional permission to eat (a core intuitive eating component) at Time 2, which subsequently predicted lower eating pathology at Time 3. Notably, only the unconditional permission to eat subscale, reflecting freedom from externally imposed food rules, mediated this relationship. Our expert skills factor may facilitate precisely this freedom by equipping individuals to evaluate which nutrition advice merits attention vs. dismissal.

The protective function of critical literacy against body image disturbance is further supported by media literacy research (43, 44). McLean et al. (43) systematically reviewed 16 studies and found that recognition of media distortion predicted lower body dissatisfaction (mean *r* = −0.24 across studies), while attention to appearance-focused content predicted higher dissatisfaction (mean *r* = 0.31). Dopelt and Houminer-Klepar (44) extended this to social media contexts, finding that food-related social media engagement significantly predicted EAT-26 scores (β = 0.23, *p* < 0.001) among Israeli college students, with body satisfaction partially mediating this relationship. Our findings suggest that expert nutrition literacy may function analogously to media literacy and may be associated with a greater ability to critically evaluate the nutrition misinformation frequently encountered by Turkish young adults on social media platforms.

The removal of SNUTLIT7 (“I know the Türkiye Dietary guidelines”) merits detailed discussion. This item demonstrated multiple psychometric weaknesses: low bootstrap stability in EGA (33.3% vs. >75% for retained items), weak corrected item-total correlation (*r* = 0.32 vs. range 0.48–0.67 for other items), and the lowest factor loading in initial CFA (41). Additionally, EGA dimensional stability analysis revealed that SNUTLIT7 inconsistently affiliated with either dimension across 1,000 bootstrap iterations, suggesting ambiguous construct membership. Beyond its statistical instability, the poor performance of SNUTLIT7 warrants careful theoretical consideration. Unlike other Information Skills items, this item assesses declarative knowledge of a specific national dietary guideline rather than applied or evaluative nutrition literacy skills. This highlights a crucial conceptual distinction: knowing that a guideline exists does not inherently mean an individual possesses the applied skills to navigate, interpret, and implement complex nutritional information in daily life. In the Turkish context, public familiarity with the Türkiye Dietary Guidelines (TÜBER) remains limited, largely due to insufficient dissemination, low visibility in public health campaigns, and minimal integration into formal education outside nutrition-related disciplines. As a result, awareness of TÜBER may reflect incidental exposure rather than an underlying competency in accessing, interpreting, or applying nutrition information. Consequently, while guideline knowledge may be an appropriate indicator of nutrition literacy in cultures with highly visible and heavily promoted national dietary frameworks (like the Flemish Food Triangle), it appears to be a less appropriate indicator in the Turkish context at present due to limited public dissemination. The original S-NutLit validation in Belgium similarly noted modest awareness of national guidelines, though item 7 performed adequately in their sample.

From a conceptual perspective, this distinction aligns with the broader differentiation between factual nutrition knowledge and functional nutrition literacy. While the latter emphasizes transferable skills such as label reading, information comparison, and critical evaluation, the former captures context-specific content knowledge that may not generalize across settings. Consequently, SNUTLIT7 appears to function as a culturally contingent indicator whose relevance depends heavily on national guideline prominence and public health infrastructure. Similar challenges may therefore arise in other countries where dietary guidelines are not widely recognized or actively promoted, suggesting that guideline-specific items should be interpreted cautiously in cross-cultural nutrition literacy research.

Importantly, Koc et al.'s (8) earlier Turkish S-NutLit adaptation (*N* = 115) also reported challenges with measurement properties, though their smaller sample and reliance on classical test theory precluded identification of the specific issues we detected. Our methodological approach—combining EGA with bootstrap stability analysis, bifactor modeling with omega indices, and propensity score matching for cross-validation—provides stronger empirical justification for item removal and establishes a more robust 10-item Turkish version.

Achieving scalar measurement invariance across both sex and BMI categories represents a critical methodological contribution enabling valid group comparisons. For sex invariance, configural (CFI ≥ 0.90, RMSEA < 0.08), metric, and scalar models demonstrated acceptable fit, with all delta values below Chen's (31) recommended cutoffs (ΔCFI < 0.010, ΔRMSEA < 0.015). This indicates that men and women interpret S-NutLit-TR items equivalently, factor loadings are comparable, and observed score differences reflect true latent differences rather than measurement artifacts (45, 46). Latent mean comparisons revealed that women scored significantly higher on expert skills (difference = 0.31, *p* < 0.01) but not on the general factor (difference = 0.08, *p* = 0.41). This pattern is consistent with Lai et al.'s (47) finding among Taiwanese college students that women demonstrated higher critical nutrition literacy despite similar functional literacy levels. Several explanations merit consideration. First, women may engage more frequently with nutrition information sources that require critical evaluation (e.g., reading food labels, consulting dietitians), developing these skills through practice. Second, societal pressures regarding body weight may paradoxically motivate women to seek expert nutrition guidance, inadvertently building critical literacy. Third, gender differences in health information-seeking behavior are well-documented, with women showing greater engagement across multiple health domains. For BMI-based invariance (underweight/normal vs. overweight/obese), scalar invariance was similarly achieved, enabling valid comparisons across weight status groups. Interestingly, latent mean differences were non-significant for both factors, suggesting that nutrition literacy levels do not differ by BMI category in our young adult sample. This contrasts with some literature reporting inverse associations between BMI and nutrition literacy in older adults, potentially reflecting cohort differences or the restricted BMI range in university populations.

From a public health perspective, the present findings underscore the importance of distinguishing between foundational nutrition literacy and more advanced expert-level competencies among young adults in Türkiye. While general nutrition literacy appears sufficient for routine dietary decisions, expert skills may be particularly critical in an information environment characterized by widespread nutrition misinformation and commercially driven health claims. Interventions targeting young adults may therefore benefit from moving beyond basic nutrition education toward strategies that enhance critical appraisal skills, media literacy, and trust calibration regarding nutrition information sources. In this respect, the S-NutLit-TR provides a theoretically grounded and psychometrically robust tool for both population surveillance and intervention evaluation.

Several limitations should be noted. The cross-sectional design does not allow causal conclusions about the proposed mediation pathway. Although the model assumes a sequence from nutrition literacy to intuitive eating and then to body appreciation, alternative directions are also plausible. For example, individuals with a more positive body image may be more inclined toward intuitive eating, which in turn could increase openness to nutrition-related information. Longitudinal designs, particularly cross-lagged panel models, are needed to clarify temporal ordering.

In addition, all variables were assessed via self-report, which raises the possibility of social desirability bias; participants may have overstated their nutrition literacy and intuitive eating tendencies while downplaying body dissatisfaction. Furthermore, all instruments were administered within a single session, which may introduce potential biases such as common method variance and response fatigue. Although this approach is widely used in survey-based research, future studies may benefit from employing multi-method or time-separated data collection designs.

The study did not include direct measures of dietary intake or diet quality, limiting the ability to interpret whether higher nutrition literacy translates into healthier dietary behaviors in practice. Future research could strengthen measurement by combining self-perceived literacy scales with objective dietary assessment tools and nutrition knowledge assessments. In addition, test–retest reliability was not assessed, limiting the evaluation of the temporal stability of the scale. Future studies should examine the stability of the S-NutLit-TR over time.

The use of snowball sampling and the focus on university students aged 18–24 further limit generalizability. This group represents a relatively homogeneous and highly educated segment of young adults, which may restrict the generalizability of the findings to broader populations, particularly non-university-attending peers who may be exposed to different nutrition information environments.

Moreover, the explained variance in body appreciation was modest (R^2^ = 0.106 in Model 1 and 0.094 in Model 2), indicating that nutrition literacy accounts for roughly 10% of the variance. While this effect is statistically significant and theoretically meaningful, it also highlights the multifactorial nature of body image, which is likely shaped more strongly by factors such as social media exposure, peer and family influences, and internalization of appearance ideals. Additionally, the mediation models presented here did not directly control for potential confounding variables such as BMI, physical activity levels, or biological sex. Although our measurement invariance tests indicated that the scale performs consistently across these groups, future research employing longitudinal designs should incorporate these variables as covariates to further clarify the nuances of the relationship between nutrition literacy, intuitive eating, and body appreciation. Finally, although the sample size (*N* = 367) exceeded common minimum recommendations for SEM and provided sufficient power for the planned analyses, larger samples would allow more reliable subgroup comparisons and improve sensitivity to smaller effects.

Future studies should address these limitations using longitudinal designs that track nutrition literacy, intuitive eating, and body appreciation across key life transitions such as university entry or early employment. Randomized controlled trials testing nutrition literacy interventions, particularly those incorporating critical evaluation and media literacy components, would provide stronger causal evidence. Validation in more diverse populations is also needed. Adolescents (13–17 years) represent a key group given the developmental sensitivity of body image during puberty; however, item content should be evaluated carefully to ensure age-appropriateness. Extending validation to older adults and clinical populations (e.g., eating disorders or obesity) would further clarify the scale's applicability across different eating-related contexts. Finally, cross-cultural studies across Mediterranean, Asian, and Anglo-American settings could determine whether the bifactor structure reflects universal or culture-specific dimensions of nutrition literacy and guide culturally sensitive nutrition education.

### 4.1 Practical implications for scoring and use of the S-NutLit-TR

The bifactor structure identified in this study has important implications for how the S-NutLit-TR should be scored and interpreted in both research and applied settings. Given that Information Skills items load almost entirely on the general factor, the total S-NutLit score can be considered a valid and parsimonious indicator of overall nutrition literacy for population-level assessments, epidemiological studies, and large-scale surveys. In such contexts, reporting only the total score may be sufficient and methodologically justified.

In contrast, the Expert Skills subscale captures meaningful variance beyond the general factor and should be reported separately when the research focus involves critical evaluation of nutrition information, susceptibility to misinformation, media literacy, or advanced decision-making competencies. For example, intervention studies aiming to improve individuals' ability to critically appraise nutrition claims, distinguish expert from non-expert sources, or interpret scientific nutrition news would benefit from emphasizing Expert Skills scores.

Discrepancies between high general nutrition literacy and lower expert skills may indicate individuals who are competent in routine nutrition-related tasks but remain vulnerable to misleading dietary messages or unsupported health claims. Conversely, high expert skills relative to general literacy may reflect selective engagement with expert information despite gaps in foundational nutrition competencies. Explicit consideration of these profiles can enhance the interpretability and practical utility of the scale across diverse research objectives.

The S-NutLit-TR holds significant utility for both clinical and public health settings. In clinical practice, dietitians can employ this brief, 10-item instrument as a rapid screening tool to identify patients with low nutrition literacy, allowing for the customization of nutritional education materials to suit their cognitive and evaluative skills. From a public health perspective, the scale provides a reliable metric for assessing the effectiveness of national nutrition campaigns and identifying vulnerable subgroups within the young adult population. Given its association with intuitive eating and body appreciation, the S-NutLit-TR can also be integrated into broader wellness programs aimed at fostering adaptive eating behaviors and positive body image, moving beyond traditional weight-centric approaches to nutrition education.

The demonstrated measurement invariance of the S-NutLit-TR across sex and BMI categories enhances its potential utility in both research and practice. Because the scale operates equivalently across these demographic groups, observed differences can be interpreted as reflecting genuine differences in nutrition literacy rather than measurement artifacts. In clinical and educational settings, the S-NutLit-TR may be used to identify individuals or groups requiring additional nutrition education support and to evaluate the effectiveness of nutrition literacy interventions. At the population level, the instrument may contribute to surveillance efforts aimed at monitoring nutrition literacy and identifying disparities among young adults. Furthermore, the scale provides a practical tool for future longitudinal and intervention studies investigating how nutrition literacy relates to dietary behaviors, health outcomes, and psychosocial constructs such as intuitive eating and body appreciation.

## 5 Conclusions

This study provides robust psychometric evidence establishing the S-NutLit-TR as a valid and reliable measure of nutrition literacy among Turkish young adults. The bifactor structure with a dominant general factor (ECV = 0.752, ω_h_ = 0.493) and a meaningful expert-specific factor (ECV = 0.248) offers a nuanced assessment framework capturing both broad nutrition literacy and specialized critical evaluation competencies. The 10-item Turkish version demonstrated scalar measurement invariance across sex and BMI groups, supporting valid comparisons in diverse research applications. The mediation findings highlight intuitive eating as a potential mechanism linking nutrition literacy to positive body image. The full mediation observed for expert skills suggests that critical evaluation competencies may be particularly important targets for intervention. Young adults equipped to critically appraise nutrition information may be better positioned to resist harmful dietary messages, maintain connection with internal eating cues, and cultivate body appreciation. These findings underscore the potential of nutrition literacy education, particularly components targeting critical evaluation and media literacy, as a strategy for promoting both healthy eating behaviors and positive body image among young adults.

## Data Availability

The raw data supporting the conclusions of this article will be made available by the authors, without undue reservation.
