# Supplementary material for: Psychometric validation of the Turkish version of the short nutrition literacy scale and its association with intuitive eating and body appreciation in young adults
Source: Front Nutr. 2026 Jul 8;13:1780768. doi: 10.3389/fnut.2026.1780768 (PMC13389934; doi:10.3389/fnut.2026.1780768)
Supplement: Supplementary file 2 [file Table_1.docx]

Supplementary Material

This Supplementary Material provides detailed psychometric analyses supporting the validation of the Turkish adaptation of the Short Nutrition Literacy Scale (S-NutLit) and the mediation model presented in the main article. The supplementary tables and figures are organized into four thematic sections that follow the analytical sequence of the study:

- Section A: Item-Level Analysis and Exploratory Network Psychometrics (Tables S1–S3),
- Section B: Confirmatory Factor Analysis and Model Selection (Table S4, Figure S1),
- Section C: Sensitivity Analysis via Propensity Score Matching (Figure S2, Tables S5–S7),
- Section D: Validity Evidence (Tables S8–S9).

All analyses were conducted using R version 4.4.2. Detailed methodological procedures are described in the main article.

**Section A: Item-Level Analysis and Exploratory Network Psychometrics**

This section documents the psychometric properties of individual S-NutLit items and the network-based dimensionality assessment that guided scale refinement [1]. The analyses presented here establish the empirical foundation for the structural decisions reported in the main article.

Table S1 presents item-level descriptive statistics including means, standard deviations, skewness, kurtosis, corrected item–total correlations, and the impact of item deletion on scale reliability. Response distributions are provided to illustrate endorsement patterns across the five-point response scale. These analyses indicated that SNUTLIT7, which assesses knowledge of the Türkiye Beslenme Rehberi (TÜBER; Turkish Dietary Guidelines), exhibited the lowest corrected item–total correlation (r = .32). Removal of this item resulted in a marginal improvement in overall scale reliability, with Cronbach’s alpha increasing from .87 to .88.

Guttman’s Lambda 6 (G6/SMC) coefficients were .90 for the total scale, .89 for the Information Skills subscale, and .75 for the Expert Skills subscale. This coefficient is calculated based on the proportion of variance in each item explained by the remaining items and provides an alternative reliability estimate to Cronbach’s alpha, particularly suitable for multidimensional scales. The mean inter-item correlations were .39 for the total scale, .48 for the Information Skills subscale, and .60 for the Expert Skills subscale. The signal-to-noise ratio was 7.0, 7.3, and 4.5, respectively, indicating high internal consistency. At the item level, corrected item–total correlations ranged from .32 to .71. The SNUTLIT7 item exhibited the lowest item–total correlation (r = .32), and removal of this item increased the overall scale alpha to .88. However, because this increase was marginal and the item was considered conceptually important, it was retained in the scale. Item skewness values ranged from −.77 to .62, and kurtosis values ranged from −.92 to −.01, indicating that the assumption of normality was met. Examination of response distributions showed that participants most frequently selected response options 3 (“undecided/sometimes”) and 4 (“agree/usually”) for the Information Skills items (SNUTLIT1–8), whereas responses for the Expert Skills items (SNUTLIT9–11) tended to cluster at lower scale points (Table S1).

Table S2 reports the results of Exploratory Graph Analysis (EGA) with 1000 bootstrap iterations, comparing the 11-item and 10-item versions of the scale. The bootstrap stability analysis examines dimension frequency, item replication rates, structural consistency, and standardized network loadings. SNUTLIT7 demonstrated critically low replication stability (33.3%) compared to all other items (72.0%–100.0%), providing robust empirical justification for its exclusion. Following item removal, the Expert Skills dimension achieved perfect structural consistency (1.000), and the confidence interval for the number of dimensions narrowed substantially.

The EGA bootstrap results indicated a stable two-dimensional structure for both the 11-item and 10-item versions of the scale. Across bootstrap samples, a two-dimensional solution was most frequently identified (69.6% for the 11-item version and 72.8% for the 10-item version), with a median of two dimensions in both cases. The reduction to a 10-item version further improved dimensional stability, as evidenced by a narrower 95% confidence interval for the number of dimensions and a marked reduction in higher-dimensional solutions. Item-level replication rates were high for all Information Skills items (SNUTLIT1–6 and SNUTLIT8), ranging from 76.6% to 99.9%, and were uniformly perfect (100%) for all Expert Skills items (SNUTLIT9–11). In contrast, SNUTLIT7 exhibited poor replication (33.3%) and was therefore classified as unstable, supporting its exclusion from the scale. Structural consistency indices corroborated the robustness of the two-factor solution, particularly in the 10-item version, in which the Expert Skills dimension achieved perfect consistency (1.000). Mean item stability was high for both dimensions and increased following item removal. Finally, standardized network loadings from the 10-item solution demonstrated that Information Skills items loaded predominantly on their intended dimension, whereas Expert Skills items showed strong and selective loadings on the Expert Skills dimension. Overall, the EGA bootstrap findings provide compelling evidence for a stable two-dimensional structure and support the psychometric refinement achieved by excluding SNUTLIT7 (Table S2).

Table S3 presents the weighted topological overlap (wTO) matrix, which assesses potential local dependence among item pairs beyond what would be expected from the latent factor structure alone. Values exceeding .25 indicate meaningful redundancy requiring consideration in model specification. The Expert Skills items exhibited the highest wTO values (.251–.379), reflecting their conceptual coherence but also suggesting potential item redundancy. These findings informed the decision to evaluate bifactor models that can accommodate such residual dependencies.

The weighted topological overlap (wTO) analysis was conducted to assess potential local dependence among items within each latent dimension. Within the Expert Skills dimension, relatively high wTO values were observed for the item pairs SNUTLIT9–SNUTLIT10 (wTO = .379) and SNUTLIT10–SNUTLIT11 (wTO = .329), indicating large redundancy and suggesting substantial shared connectivity beyond what would be expected from the latent factor alone. The pair SNUTLIT9–SNUTLIT11 also demonstrated moderate-to-large redundancy (wTO = .251), further supporting the presence of strong inter-item dependence within this subscale. In the Information Skills dimension, several item pairs exhibited moderate redundancy, including SNUTLIT6–SNUTLIT8 (wTO = .281), SNUTLIT2–SNUTLIT3 (wTO = .278), and SNUTLIT3–SNUTLIT4 (wTO = .262), indicating meaningful but less pronounced local dependence. Additional item pairs showed small redundancy (wTO range = .172–.198), reflecting limited shared variance beyond the general factor. Overall, the wTO findings suggest that local dependence is most pronounced within the Expert Skills dimension, consistent with its conceptual coherence but also indicating potential item redundancy. In contrast, the Information Skills dimension demonstrates more distributed and modest local dependence, supporting its broader and more heterogeneous content coverage. These results provide empirical justification for evaluating bifactor or network-informed modeling approaches to appropriately account for residual item dependencies in subsequent analyses (Table S3).

**Section B: Confirmatory Factor Analysis and Model Selection**

This section presents the systematic evaluation of alternative factor structures and documents the rationale for selecting the final bifactor model.

Table S4 compares the fit indices and factor loadings across four competing models: (1) one-factor, (2) two-factor oblique, (3) two-factor orthogonal, and (4) bifactor with both Information-specific and Expert-specific factors. The one-factor model showed poor fit (RMSEA = .229, SRMR = .148), indicating that the items cannot be adequately represented by a single latent dimension. The two-factor orthogonal model performed even worse than the one-factor model, demonstrating that the Information Skills and Expert Skills dimensions share meaningful common variance and cannot be treated as independent. The two-factor oblique model showed excellent fit (CFI = .996, RMSEA = .069), supporting the presence of two correlated factors. However, the full bifactor model achieved superior fit with a non-significant chi-square test (p = .156), and revealed a critical pattern: Information Skills items loaded strongly on the general factor (.655–.915) but showed weak and mostly non-significant loadings on the Information-specific factor (−.303 to .144). In contrast, Expert Skills items loaded significantly on both the general factor (.295–.409) and the Expert-specific factor (.651–.846). This pattern indicated that the Information Skills items primarily represent general nutrition literacy, while Expert Skills capture unique variance beyond the general construct. Accordingly, the final model retained only the General Factor and the Expert-specific factor.

Examination of the model fit indices indicated that the one-factor model did not provide an adequate fit to the data. Although the CFI value (.956) approached the acceptable range, both the RMSEA (.229) and SRMR (.148) values substantially exceeded recommended thresholds. This finding suggests that the 10 items cannot be adequately represented by a single general nutrition literacy factor. The two-factor orthogonal model, which assumes no correlation between the latent factors, demonstrated even poorer fit than the one-factor model. This result indicates that the Information Skills and Expert Skills factors are not independent and share meaningful common variance. In contrast, the two-factor oblique model showed excellent fit to the data, with CFI (.996) and TLI (.995) values exceeding the .95 criterion, an acceptable RMSEA (.069), and a good SRMR (.050). These findings support the presence of two correlated dimensions underlying the scale. The bifactor model yielded the best overall fit. The chi-square test was non-significant (*p* = .156), indicating excellent model–data fit. In the bifactor model, all items load simultaneously on a general factor and on their respective specific factors. Inspection of the bifactor loadings revealed a critical pattern: the Information Skills items (SNUTLIT1–8) loaded strongly and significantly on the general factor (.655–.915), whereas their loadings on the Information-specific factor were small (ranging from −.303 to .144) and mostly non-significant. In contrast, the Expert Skills items (SNUTLIT9–11) loaded significantly on both the general factor (.295–.409) and the Expert-specific factor (.651–.846), with substantially higher loadings on the specific factor. This pattern indicates that the variance of the Information Skills items is largely explained by the general factor and does not constitute a distinct specific dimension. In other words, Information Skills items primarily represent general nutrition literacy (Table S4).

Expert Skills items, however, contribute both to the general construct and to a distinct source of variance beyond the general factor. This finding is theoretically meaningful, as Expert Skills reflect advanced competencies—such as evaluating nutrition information sources, distinguishing nutrition experts, and critically appraising nutrition-related news—that build upon, yet remain distinct from, basic nutrition literacy. Accordingly, the final bifactor model excluded the Information Skills specific factor and retained a structure consisting of a General Factor and an Expert Skills specific factor.

Figure S1 displays the path diagram of the final bifactor model with standardized factor loadings. Arrow thicknesses are proportional to loading magnitudes, visually illustrating the dominant role of the general factor for Information Skills items and the dual contribution of both factors for Expert Skills items.

Figure S1 presents the path diagram of the final bifactor model. As shown, the General Factor loads on all 10 items, with standardized loadings ranging from .295 to .910. The Information Skills items (SNUTLIT1–8) show strong loadings on the general factor (.650–.910), whereas the Expert Skills items exhibit moderate loadings on the general factor (.295–.408). The Expert Skills specific factor loads exclusively on SNUTLIT9, SNUTLIT10, and SNUTLIT11, with standardized loadings ranging from .651 to .847. The correlation between the General Factor and the Expert-specific factor was fixed to zero, satisfying the orthogonality assumption of bifactor models. Arrow thicknesses are proportional to the magnitude of standardized loadings, visually highlighting the strong association between Information Skills items and the general factor, as well as the dual association of Expert Skills items with both the general and specific factors. The final bifactor model demonstrated excellent fit (CFI = .997, TLI = .996, RMSEA = .062, SRMR = .043). Examination of factor loadings confirmed that all items loaded significantly on the general factor. Information Skills items loaded exclusively on the general factor (.650–.910), whereas Expert Skills items loaded on both the general factor (.295–.408) and the Expert-specific factor (.651–.847). Communality values (h²) ranged from .423 to .828, indicating that a substantial proportion of item variance was explained by the model, with the highest communalities observed for SNUTLIT3 (.828) and SNUTLIT10 (.804).

**Section C: Sensitivity Analysis via Propensity Score Matching**

A key methodological challenge in scale validation studies with limited sample sizes is the need to conduct both exploratory and confirmatory analyses. When the same data are used for both purposes, confirmatory analyses may be biased toward "confirming" structures that were derived from that same dataset. To address this limitation, propensity score matching (PSM) was employed to create two independent subsamples that are demographically equivalent, allowing for genuine cross-validation of the factor structure.

Figure S2 illustrates the propensity score distributions before and after matching. The matching procedure used a nearest-neighbor algorithm with 1:1 matching ratio and a caliper of 0.25 standard deviations to ensure high-quality matches.

Propensity score matching was conducted using a nearest-neighbor algorithm with a 1:1 matching ratio, based on propensity scores estimated via a generalized linear model (GLM). A caliper of 0.25 standard deviations was applied to restrict matches to individuals with sufficiently similar propensity scores, thereby reducing the risk of poor-quality matches. Prior to matching, the analytic sample comprised 366 participants. After matching, two well-balanced subsamples of 170 participants each were obtained for the EFA and CFA analyses, supporting the use of independent but comparable samples for cross-validation. As a consequence of the caliper restriction, 26 participants remained unmatched, reflecting an acceptable trade-off between sample size and improved covariate balance across the matched groups (Figure S2).

Table S5 presents comprehensive balance diagnostics for the matched subsamples. All standardized mean differences (SMD) for continuous variables were below .10 (range: 0.011–0.061), indicating excellent balance. Statistical tests (t-tests for continuous variables, χ² tests for categorical variables) confirmed no significant differences between groups across all 10 matching covariates (all p > .05). The final matched sample comprised 170 participants in each subsample (EFA and CFA groups), with 26 participants remaining unmatched due to the caliper restriction.

Statistical tests further confirmed the absence of significant differences between the groups. Independent-samples *t*-tests applied to continuous variables (age: *t* = −0.11, *p* = .916; height: *t* = 0.47, *p* = .638; weight: *t* = 0.56, *p* = .573) and chi-square tests applied to categorical variables (sex: χ² = 0.01, *p* = .907; education: χ² = 0.00, *p* = 1.000; income: χ² = 1.34, *p* = .510; chronic disease: χ² = 0.00, *p* = 1.000; medical nutrition therapy: χ² = 0.00, *p* = 1.000; nutrition education: χ² = 0.05, *p* = .823; physical activity: χ² = 0.68, *p* = .878) yielded non-significant results (*p* > .05 for all comparisons), indicating that the groups were statistically equivalent across all examined variables. Following this balance assessment, a robustness-oriented analytic strategy was adopted by proceeding with target-rotated exploratory factor analysis (EFA) and confirmatory factor analysis (CFA) using the matched subsamples. The EFA group (*n* = 170) was used for the target-rotated factor analysis, whereas the CFA group (*n* = 170) was employed to validate the bifactor model. This cross-validation approach enabled the factor structure to be tested in independent samples, thereby strengthening the psychometric validity of the study (Table S5).

Table S6 reports the results of target-rotated exploratory factor analysis and Schmid–Leiman bifactor decomposition conducted in the EFA subsample (n = 170). The target rotation, guided by the hypothesized bifactor structure, confirmed that all items loaded strongly on the general factor (.772–1.006) while only Expert Skills items loaded meaningfully on the Expert-specific factor (.895–1.134). The Schmid–Leiman solution provided orthogonalized loadings and revealed that an Information-specific factor also emerged with moderate loadings (.49–.67) in this subsample, suggesting potential three-factor representation at the exploratory level. Reliability indices from this solution indicated high total reliability (ω_t_ = .93), moderate general factor reliability (ω_h_ = .51), and acceptable specific factor reliabilities (ω_g_ = .49–.54). Model fit was acceptable for exploratory purposes (RMSEA = .092, TLI = .938).

The target-rotated exploratory factor analysis (EFA) largely supported the a priori hypothesized structure. All items loaded strongly on the general factor, with loadings ranging from .772 to 1.006, indicating the presence of a dominant overarching nutrition literacy construct. The highest general factor loadings were observed for SNUTLIT3 (.950), SNUTLIT4 (.961), SNUTLIT9 (.989), SNUTLIT10 (1.006), and SNUTLIT11 (.969). Loadings exceeding unity are permissible in oblique rotations and reflect correlations among latent factors rather than estimation artifacts. A critical finding of the target-rotated solution concerns the Expert Skills factor, on which only the theoretically designated expert-related items loaded meaningfully: SNUTLIT9 (.982), SNUTLIT10 (1.134), and SNUTLIT11 (.895). In contrast, the Information Skills items showed negligible and practically insignificant loadings on this factor (range: −.074 to .192). This loading pattern closely matches the predefined target matrix and provides strong support for the bifactor model assumptions (Table S6).

The Schmid–Leiman decomposition further clarified the bifactor structure by orthogonalizing the general and specific factors and constraining loadings within the standardized range. In this solution, loadings on the general factor were moderate and relatively homogeneous across items (range: .46–.58), indicating a stable and evenly distributed general construct. Information Skills items loaded on the Information-specific factor with coefficients between .49 and .67, whereas Expert Skills items loaded on the Expert-specific factor with coefficients ranging from .54 to .69. Notably, unlike the full-sample CFA results, the Information-specific factor emerged with meaningful loadings in the EFA subsample, suggesting that this dimension may be more detectable under the flexible assumptions of EFA or due to subsample-specific characteristics. Reliability indices derived from the Schmid–Leiman solution provide additional insight into the measurement properties of the scale. The omega total (ωt = .93) indicates excellent overall reliability of the total score. The omega hierarchical (ωh = .51) suggests that approximately half of the reliable variance in the total score is attributable to the general factor. The omega group coefficients for the Information (ωg = .54) and Expert (ωg = .49) factors indicate that, even after accounting for the general factor, the specific dimensions retain a moderate amount of reliable unique variance. Model fit indices were acceptable for exploratory purposes. The RMSEA of .092 (90% CI [.064, .122]) is slightly above the conventional .08 threshold but remains within acceptable limits for EFA (< .10). The TLI (.938) exceeds the .90 criterion, the χ²/df ratio (2.45) falls well below the upper acceptable limit, and the RMSR (.03) indicates good correspondence between the model and observed correlations. Together, the two-factor solutions explained a substantial proportion of variance (65.3% for the target-rotated solution and 64.0% for the Schmid–Leiman solution). In the target-rotated EFA, the general factor alone accounted for 57.4% of the variance, whereas in the Schmid–Leiman solution this proportion decreased to 27.0% as variance was redistributed across the general and specific factors. Communalities were consistent across solutions, ranging from .46 to .78, with the highest values observed for SNUTLIT3, SNUTLIT10, and SNUTLIT4 (Table S6).

Overall, these findings demonstrate that both the target-rotated EFA and the Schmid–Leiman bifactor solution provide strong evidence for the multidimensional validity of the scale in the PSM-derived independent EFA subsample (*n* = 170). All items loaded significantly on the general factor, while Expert Skills items additionally loaded on a specific factor. The emergence of an Information-specific factor in the Schmid–Leiman solution suggests a potential three-factor representation (general + information-specific + expert-specific) at the exploratory level. Nevertheless, the moderate omega group values and the consistent presence of a strong general factor support the interpretability and practical use of the total score. Based on these results, the bifactor structure identified in the EFA subsample was subsequently subjected to confirmatory factor analysis in an independent subsample (*n* = 170), with the final model specification focusing on the general factor and the Expert-specific factor.

Table S7 presents the cross-validation CFA results from the independent CFA subsample (n = 170). The final bifactor model (General + Expert-specific) demonstrated good fit (CFI = .993, TLI = .990, RMSEA = .077, SRMR = .065). Factor loadings closely replicated those from the full sample, with Information Skills items loading .575–.911 on the general factor and Expert Skills items showing the expected bifactor pattern (general: .181–.394; Expert-specific: .602–.874). Bifactor indices were consistent with full-sample results: ECV = .731 (vs. .752 in full sample), H indices exceeded .80 for both factors, and ωh = .429 supported the use of both total and subscale scores.

In the PSM-derived independent CFA subsample (*n* = 170), the bifactor model demonstrated a good overall fit to the data. Examination of the model fit indices showed that both the CFI (.993) and TLI (.990) exceeded the conventional criterion for excellent fit (.95). The RMSEA value (.077), with a 90% confidence interval of [.049, .104], fell below the acceptable threshold of .08, and the SRMR (.065) was also below the recommended cutoff, further supporting adequate model fit. The relative chi-square statistic (χ²/df = 2.00) indicated good fit according to commonly accepted standards (< 3) (Table S7).

Inspection of the standardized factor loadings revealed that the Information Skills items (SNUTLIT1–SNUTLIT8) loaded strongly on the general factor, with coefficients ranging from .575 to .911. The highest loadings were observed for SNUTLIT3 (.911), SNUTLIT2 (.844), and SNUTLIT4 (.841). Although SNUTLIT1 (.575) and SNUTLIT5 (.602) showed comparatively lower loadings, all general-factor loadings for these items were statistically significant (*p* < .001), indicating robust contributions to the overall construct. For the Expert Skills items (SNUTLIT9–SNUTLIT11), the bifactor structure was clearly evident. These items loaded on both the general factor (range: .181–.394) and the Expert-specific factor (range: .602–.874). Importantly, the loadings on the Expert-specific factor were substantially higher than those on the general factor. In particular, SNUTLIT10 exhibited the strongest Expert-specific loading (.874) while showing a relatively weak general-factor loading (.181). This pattern confirms that the Expert Skills items capture meaningful variance beyond general nutrition literacy, supporting their conceptual distinctiveness (Table S7).

Bifactor indices provided further evidence regarding the structure and reliability of the model. The ECV value (.731) indicated that 73.1% of the common variance was explained by the general factor, consistent with the ECV obtained in the full sample (.752). The H indices exceeded the .80 threshold for both the general factor (.933) and the Expert-specific factor (.823), suggesting that both constructs are well defined and likely to be replicable across samples. The omega hierarchical coefficient (ω_h_ = .429) indicated that approximately 43% of the reliable variance in the total score was attributable to the general factor. Although slightly lower than the corresponding value in the full sample (.493), this finding supports the reporting of both total and subscale scores. The omega group coefficient for the Expert factor (ω_g_ = .527) demonstrated that the Expert-specific factor retained a reliable amount of unique variance even after controlling for the general factor (Table S7).

Internal consistency estimates were satisfactory, with Cronbach’s α values of .838 for the total scale, .867 for the Information Skills subscale, and .758 for the Expert Skills subscale, all exceeding the minimum acceptable threshold of .70. Communality estimates ranged from .331 to .830, with the highest values observed for SNUTLIT3 (.830), SNUTLIT10 (.797), and SNUTLIT2 (.712). Although SNUTLIT1, SNUTLIT5, and SNUTLIT11 exhibited comparatively lower communalities, their statistically significant factor loadings indicate that these items still make meaningful contributions to the latent structure. Overall, the CFA results obtained from the PSM-derived subsample provide strong support for the proposed bifactor model, confirming the presence of a dominant general nutrition literacy factor alongside a distinct and reliable Expert Skills factor (Table S7).

**Section D: Validity Evidence**

This section provides comprehensive validity evidence including convergent, discriminant, and known-groups validity.

Table S8 presents the complete correlation matrix among S-NutLit scores (General and Expert factors), Body Appreciation Scale (BAS), and Intuitive Eating Scale-2 (IES-2) scores. The strong correlation between S-NutLit General and Expert scores (r = .673) confirms that these factors measure related but distinct aspects of nutrition literacy. Convergent validity was supported by significant positive correlations between S-NutLit General and both BAS (r = .236) and IES-2 (r = .416). The Expert factor showed weaker but significant correlations with these criterion measures (BAS: r = .114; IES-2: r = .186), consistent with its more specialized focus on critical evaluation skills.

Descriptive statistics indicated that participants demonstrated a moderate level of overall nutrition literacy, with a mean S-NutLit General score of 3.22 (SD = 0.80) on a five-point scale. In contrast, the mean score for the Expert-specific factor was noticeably lower (M = 2.68, SD = 1.00), suggesting that competencies related to evaluating expert sources and critically appraising nutrition information are less developed than general, functional nutrition literacy skills in this sample. Median values closely mirrored the means for both factors, and skewness and kurtosis values fell within acceptable ranges, supporting approximate normality of the score distributions. Body Appreciation and Intuitive Eating scores were relatively high (M = 3.53 and M = 3.20, respectively), indicating generally positive body-related attitudes and adaptive eating behaviors in the study population. The Intuitive Eating score showed a moderately negative skewness (−0.804) and elevated kurtosis (1.476), reflecting a tendency toward higher endorsement levels and some clustering at the upper end of the scale (Table S8).

Correlation analyses further supported the construct validity of the S-NutLit scale. A strong positive association was observed between the S-NutLit General and Expert factor scores (r = .673, p < .001), indicating that while the two dimensions are closely related, they remain empirically distinguishable components of the broader nutrition literacy construct. In terms of convergent validity, the General factor demonstrated significant positive correlations with both Body Appreciation (r = .236, p < .001) and Intuitive Eating (r = .416, p < .001). The latter association was of moderate magnitude, suggesting that higher overall nutrition literacy is meaningfully linked to more adaptive, internally regulated eating behaviors. By contrast, the Expert-specific factor exhibited weaker yet statistically significant correlations with Body Appreciation (r = .114, p = .029) and Intuitive Eating (r = .186, p < .001). This attenuated pattern implies that Expert Skills reflect a more specialized form of nutrition-related competence—focused on critical evaluation of professional information—rather than everyday eating attitudes or behaviors. Taken together, the observed mean differences and correlation patterns reinforce the conceptual distinction between general and expert nutrition literacy while providing further evidence for the scale’s convergent and discriminant validity (Table S8).

Table S9 provides comprehensive known-groups validity analyses across all sociodemographic characteristics for all four scale scores. For the S-NutLit General factor, significantly higher scores were observed among individuals with nutrition education (t = 5.23, p < .001), medical nutrition therapy experience (t = 3.47, p < .001), and regular meal patterns (F = 2.77, p = .041). For the S-NutLit Expert factor, significant differences emerged for sex (females > males; t = −2.14, p = .033), nutrition education (t = 6.18, p < .001), medical nutrition therapy (t = 3.82, p < .001), and physical activity level (F = 3.93, p = .009). Body appreciation scores differed significantly by income status and BMI classification, while intuitive eating scores showed no significant group differences. These patterns demonstrate that the S-NutLit successfully discriminates between groups expected to differ in nutrition literacy based on their experiences and behaviors.

The S-NutLit General factor score was significantly higher among individuals who had received nutrition education compared with those who had not (*t* = 5.23, *p* < .001), and a similar pattern was observed for individuals who had undergone medical nutrition therapy (*t* = 3.47, *p* < .001). When examined by meal frequency, participants consuming two or three meals per day exhibited higher general nutrition literacy scores than those consuming only one meal per day (*F* = 2.77, *p* = .041). With respect to the S-NutLit Expert factor score, females scored significantly higher than males (*t* = −2.14, *p* = .033). Higher Expert factor scores were also observed among individuals who had received nutrition education (*t* = 6.18, *p* < .001), those who had undergone medical nutrition therapy (*t* = 3.82, *p* < .001), and those engaging in physical activity compared with inactive individuals (*F* = 3.93, *p* = .009). Body Appreciation Scale scores differed significantly by income status (*F* = 6.45, *p* = .002) and BMI classification (*F* = 10.06, *p* < .001). Participants whose income exceeded their expenses reported higher body appreciation than other income groups, while individuals classified as underweight or normal weight reported higher body appreciation compared with those classified as overweight or obese (Table S9).

No statistically significant group differences were observed with respect to education level, presence of chronic disease, or Intuitive Eating Scale scores (*p* > .05). Overall, these findings indicate that the S-NutLit scale successfully discriminates between individuals with differing nutrition-related experiences and behaviors, thereby providing strong support for the discriminant validity of the scale (Table S9).

**References**

1. Christensen, A.P.; Golino, H. On the Equivalency of Factor and Network Loadings. *Behav Res Methods* 2021, *53*, 1563–1580, doi:10.3758/s13428-020-01500-6.

2. Golino, H.F.; Epskamp, S. Exploratory Graph Analysis: A New Approach for Estimating the Number of Dimensions in Psychological Research. *PLoS One* 2017, *12*, e0174035, doi:10.1371/journal.pone.0174035.

3. Christensen, A.P.; Garrido, L.E.; Golino, H. Unique Variable Analysis: A Network Psychometrics Method to Detect Local Dependence. *Multivariate Behav Res* 2023, *58*, 1165–1182, doi:10.1080/00273171.2023.2194606.

**Supplementary Tables**

Table S1. Item-Level Descriptive Statistics and Response Distributions (N = 367)

| **Item / Scale** | **Content** | | **Mean ± SD** | | **Skewness** | | **Kurtosis** | | **SE** | **Item-Total r** | | **Alpha if Item Deleted** | |
| --- | --- | --- | --- | --- | --- | --- | --- | --- | --- | --- | --- | --- | --- |
| SNUTLIT1 | Understanding food labels | | 3.28 ± 1.09 | | −0.43 | | −0.45 | | 0.06 | 0.54 | | 0.86 | |
| SNUTLIT2 | Comparing nutritional values | | 3.57 ± 1.16 | | −0.75 | | −0.23 | | 0.06 | 0.68 | | 0.86 | |
| SNUTLIT3 | Using portion information | | 3.58 ± 1.09 | | −0.77 | | −0.01 | | 0.06 | 0.71 | | 0.85 | |
| SNUTLIT4 | Understanding daily reference values | | 3.37 ± 1.15 | | −0.47 | | −0.57 | | 0.06 | 0.70 | | 0.85 | |
| SNUTLIT5 | Calculating calories | | 3.47 ± 1.17 | | −0.52 | | −0.55 | | 0.06 | 0.65 | | 0.86 | |
| SNUTLIT6 | Making healthy choices | | 3.47 ± 1.16 | | −0.62 | | −0.40 | | 0.06 | 0.70 | | 0.85 | |
| SNUTLIT7 | Knowing TUBER guidelines | | 2.20 ± 1.22 | | 0.62 | | −0.81 | | 0.06 | 0.32 | | 0.88 | |
| SNUTLIT8 | Evaluating food content | | 3.44 ± 1.21 | | −0.54 | | −0.58 | | 0.06 | 0.64 | | 0.86 | |
| SNUTLIT9 | Evaluating nutrition experts | | 2.88 ± 1.08 | | −0.02 | | −0.72 | | 0.06 | 0.52 | | 0.87 | |
| SNUTLIT10 | Evaluating nutrition information sources | | 2.47 ± 1.20 | | 0.37 | | −0.81 | | 0.06 | 0.44 | | 0.87 | |
| SNUTLIT11 | Critically reading nutrition news | | 2.68 ± 1.24 | | 0.20 | | −0.92 | | 0.06 | 0.48 | | 0.87 | |
| **Subscale** | **Information Skills (**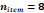**)** | **3.30 ± 0.85** | | – | | **α** = 0.88 (%95 GA: 0.86-0.90) | | | | | | | |
| **Subscale** | **Expert Skills (**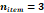**)** | **2.70 ± 1.00** | | – | | **α** = 0.82 (%95 GA: 0.78-0.85) | | | | | | | |
| **Scale** | **S-NutLit Total (**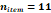**)** | **3.10 ± 0.77** | | – | | **α** = 0.87 (%95 GA: 0.85-0.89) | | | | | | | |
|  | **Item Code** | **1 (%)** | | **2 (%)** | | **3 (%)** | | **4 (%)** | | | **5 (%)** | |  |
|  | SNUTLIT1 | 8 | | 14 | | 31 | | 36 | | | 11 | |  |
|  | SNUTLIT2 | 8 | | 11 | | 18 | | 44 | | | 20 | |  |
|  | SNUTLIT3 | 6 | | 10 | | 20 | | 46 | | | 18 | |  |
|  | SNUTLIT4 | 8 | | 14 | | 25 | | 37 | | | 16 | |  |
|  | SNUTLIT5 | 8 | | 13 | | 23 | | 36 | | | 20 | |  |
|  | SNUTLIT6 | 8 | | 11 | | 22 | | 40 | | | 18 | |  |
|  | SNUTLIT7 | 40 | | 23 | | 19 | | 15 | | | 4 | |  |
|  | SNUTLIT8 | 10 | | 12 | | 23 | | 35 | | | 20 | |  |
|  | SNUTLIT9 | 11 | | 26 | | 33 | | 24 | | | 6 | |  |
|  | SNUTLIT10 | 27 | | 25 | | 28 | | 14 | | | 6 | |  |
|  | SNUTLIT11 | 22 | | 22 | | 30 | | 17 | | | 9 | |  |
|  |  |  |  |  |  |  |  |  |  |  |  |  |  |

Standardized Cronbach’s α: total = 0.87, Information = 0.88, Expert = 0.82; G6 (SMC): total = 0.90, Information = 0.89, Expert = 0.75; mean inter-item correlation (r): total = 0.39, Information = 0.48, Expert = 0.60; signal-to-noise ratio (S/N): total = 7.0, Information = 7.3, Expert = 4.5. SD: standard deviation; SE: standard error; Item–total r: corrected item–total correlation. Items 1, 9, 10, and 11 use a frequency response format (1 = Never to 5 = Always), whereas the remaining items use an agreement response format (1 = Strongly disagree to 5 = Strongly agree). SNUTLIT7 was subsequently removed due to poor psychometric properties.

Table S2. EGA Bootstrap Stability Analysis Results (1000 Iterations)

| **Structure** | **11-Item Version** | **10-Item Version** | **Item** | **11-Item Version** | **10-Item Version** | **Assigned Dimension** | **Information Skills** | **Expert Skills** |
| --- | --- | --- | --- | --- | --- | --- | --- | --- |
| 1 dimension | 0.0% | 0.0% | SNUTLIT1 | 72.0% | 79.7% | Information Skills | .249 | .031 |
| 2 dimensions | 69.6% | 72.8% | SNUTLIT2 | 99.6% | 99.8% | Information Skills | .492 | .058 |
| 3 dimensions | 24.7% | 27.1% | SNUTLIT3 | 99.6% | 99.9% | Information Skills | .687 | .072 |
| 4 dimensions | 5.7% | 0.1% | SNUTLIT4 | 99.7% | 99.8% | Information Skills | .509 | .089 |
| Median dimensions | 2 | 2 | SNUTLIT5 | 76.6% | 78.5% | Information Skills | .374 | .068 |
| 95% CI | [0.85, 3.15] | [1.12, 2.88] | SNUTLIT6 | 82.0% | 85.3% | Information Skills | .565 | .042 |
| **Dimension** |  |  | SNUTLIT7 | 33.3% | **–** | Unstable | – | – |
| Information Skills | 0.685 | 0.728 | SNUTLIT8 | 82.0% | 84.2% | Information Skills | .415 | .037 |
| Expert Skills | 0.588 | 1.000 | SNUTLIT9 | 100.0% | 100.0% | Expert Skills | .065 | .536 |
| Mean Item Stability *(Information)* | 87.4% | 89.6% | SNUTLIT10 | 100.0% | 100.0% | Expert Skills | .048 | .653 |
| Mean Item Stability *(Expert)* | 83.3% | 100.0% | SNUTLIT11 | 100.0% | 100.0% | Expert Skills | .057 | .468 |

The 10-item version refers to the model in which SNUTLIT7 was removed. Bootstrap analyses were conducted using 1000 parametric iterations with GLASSO network estimation and Walktrap community detection [2]. Dimension frequency indicates the proportion of bootstrap samples in which a given number of dimensions was identified. Replication rate represents the percentage of bootstrap samples in which an item was assigned to its primary dimension. Structural consistency reflects the proportion of bootstrap samples reproducing the exact item composition of each dimension. Standardized network loadings are reported for the 10-item solution.

Table S3. Weighted Topological Overlap (wTO) Matrix for Local Dependence Assessment

| **Item Pair** | **wTO** | **Interpretation** |
| --- | --- | --- |
| *Expert Skills Dimension* |  |  |
| SNUTLIT9-SNUTLIT10 | .379 | Large redundancy |
| SNUTLIT10-SNUTLIT11 | .329 | Large redundancy |
| SNUTLIT9-SNUTLIT11 | .251 | Moderate-large redundancy |
| *Information Skills Dimension* |  |  |
| SNUTLIT6-SNUTLIT8 | .281 | Moderate redundancy |
| SNUTLIT2-SNUTLIT3 | .278 | Moderate redundancy |
| SNUTLIT3-SNUTLIT4 | .262 | Moderate redundancy |
| SNUTLIT4-SNUTLIT5 | .198 | Small redundancy |
| SNUTLIT2-SNUTLIT4 | .185 | Small redundancy |
| SNUTLIT3-SNUTLIT6 | .172 | Small redundancy |

wTO = Weighted Topological Overlap. Interpretation based on Christensen, Garrido, and Golino [3]: wTO > .25 indicates potential local dependence requiring attention. Only item pairs with wTO > .15 are shown. High wTO values among Expert Skills items reflect the coherent but potentially redundant nature of this subscale.

Tablo S4. Confirmatory Factor Analysis Results for Alternative Measurement Models

| **Fit Index** | | **One Factor** | | | **Two-Factor (Oblique)** | | **Two-Factor (Orthogonal)** | | | **Bifactor** |
| --- | --- | --- | --- | --- | --- | --- | --- | --- | --- | --- |
| χ² | | 708.29 | | | 93.17 | | 795.45 | | | 32.06 |
| sd | | 35 | | | 34 | | 35 | | | 25 |
| p | | <.001 | | | <.001 | | <.001 | | | .156 |
| CFI | | .956 | | | .996 | | .951 | | | 1.000 |
| TLI | | .944 | | | .995 | | .937 | | | .999 |
| RMSEA | | .229 | | | .069 | | .244 | | | .028 |
| RMSEA %90 GA | | [.215, .244] | | | [.052, .086] | | [.229, .258] | | | [.000, .053] |
| SRMR | | .148 | | | .050 | | .185 | | | .035 |
| **Item** | **Factor** | **λ** | **SE** | **p** | | **General Factor (λ_G_)** | | **Information-Specific (λ_S1_)** | **Expert-Specific (λ_S2_)** | |
| SNUTLIT1 | Information | .650 | .029 | <.001 | | .655*** | | .116 | — | |
| SNUTLIT2 | Information | .845 | .017 | <.001 | | .849*** | | .144 | — | |
| SNUTLIT3 | Information | .910 | .011 | <.001 | | .915*** | | .136 | — | |
| SNUTLIT4 | Information | .855 | .014 | <.001 | | .857*** | | .098 | — | |
| SNUTLIT5 | Information | .728 | .024 | <.001 | | .717*** | | –.237* | — | |
| SNUTLIT6 | Information | .845 | .015 | <.001 | | .842*** | | –.303* | — | |
| SNUTLIT8 | Information | .769 | .021 | <.001 | | .755*** | | –.296* | — | |
| SNUTLIT9 | Expert | .846 | .026 | <.001 | | .409*** | | — | .697*** | |
| SNUTLIT10 | Expert | .813 | .025 | <.001 | | .295*** | | — | .846*** | |
| SNUTLIT11 | Expert | .783 | .028 | <.001 | | .380*** | | — | .651*** | |
|  |  |  |  |  |  |  |  |  |  |  |

λ: standardized factor loading; λ_G_: general factor loading; λ_S1_: Information Skills specific factor loading; λ_S2_: Expert Skills specific factor loading; SE: standard error; CI: confidence interval. The WLSMV estimator was used. *p* < .05, ***p* < .001.

Table S5. Propensity Score Matching Balance Diagnostics

| **Variable** | **SMD** | **EFA Group (n=170)** | **CFA Group (n=170)** | **t** | **p** |
| --- | --- | --- | --- | --- | --- |
| Age (years) | 0.011 | 20.91 ± 2.07 | 20.94 ± 2.03 | -0.11 | .916 |
| Height (cm) | -0.051 | 169.21 ± 9.16 | 168.55 ± 9.24 | 0.47 | .638 |
| Weight (kg) | -0.061 | 67.43 ± 17.87 | 66.34 ± 16.96 | 0.56 | .573 |
| **Variable** | **Category** | **EFA Group n (%)** | **CFA Group n (%)** | **χ²** | **p** |
| Sex | Male | 54 (31.8%) | 53 (31.2%) | 0.01 | .907 |
|  | Female | 116 (68.2%) | 117 (68.8%) |  |  |
| Education | Below undergraduate | 46 (27.1%) | 46 (27.1%) | 0.00 | 1.000 |
|  | Undergraduate+ | 124 (72.9%) | 124 (72.9%) |  |  |
| Income | Below expenses | 45 (26.5%) | 39 (22.9%) | 1.34 | .510 |
|  | Equal to expenses | 87 (51.2%) | 96 (56.5%) |  |  |
|  | Above expenses | 38 (22.4%) | 35 (20.6%) |  |  |
| Chronic disease | Yes | 15 (8.8%) | 15 (8.8%) | 0.00 | 1.000 |
|  | No | 155 (91.2%) | 155 (91.2%) |  |  |
| Medical nutrition therapy | Yes | 8 (4.7%) | 8 (4.7%) | 0.00 | 1.000 |
|  | No | 162 (95.3%) | 162 (95.3%) |  |  |
| Nutrition education | Yes | 65 (38.2%) | 63 (37.1%) | 0.05 | .823 |
|  | No | 105 (61.8%) | 107 (62.9%) |  |  |
| Physical activity | None | 34 (20.0%) | 37 (21.8%) | 0.68 | .878 |
|  | Light | 79 (46.5%) | 80 (47.1%) |  |  |
|  | Moderate | 38 (22.4%) | 33 (19.4%) |  |  |
|  | Vigorous | 19 (11.2%) | 20 (11.8%) |  |  |

SMD: Standardized Mean Difference. All SMD values < 0.10 indicate excellent balance. All statistical tests non-significant (p > .05), confirming successful matching.

Table S6. Target-Rotated EFA and Schmid–Leiman Bifactor EFA Results (n_EFA_ = 170)

| **Item** | **Target-Rotated EFA Model** | | | **Schmid–Leiman Bifactor EFA** | | | |
| --- | --- | --- | --- | --- | --- | --- | --- |
|  | **λ_General_** | **λ_Expert_** | **h²** | **λ_General_** | **λ_Information_** | **λ_Expert_** | **h²** |
| **SNUTLIT1** | **.772** | .158 | .457 | .46 | .49 | — | .46 |
| **SNUTLIT2** | **.848** | .083 | .631 | .51 | .61 | — | .63 |
| **SNUTLIT3** | **.950** | .099 | .785 | .57 | .67 | — | .78 |
| **SNUTLIT4** | **.961** | .161 | .742 | .58 | .64 | — | .74 |
| **SNUTLIT5** | **.868** | .192 | .566 | .52 | .54 | — | .57 |
| **SNUTLIT6** | **.832** | .007 | .685 | .51 | .65 | — | .68 |
| **SNUTLIT8** | **.776** | −.074 | .684 | .48 | .67 | — | .68 |
| **SNUTLIT9** | .989 | **.982** | .638 | .53 | — | .59 | .64 |
| **SNUTLIT10** | 1.006 | **1.134** | .766 | .53 | — | .69 | .77 |
| **SNUTLIT11** | .969 | **.895** | .575 | .53 | — | .54 | .57 |
| **SS Loadings** | 5.74 | 0.79 | — | 2.70 | 2.60 | 1.10 | — |
| **EVP** | %57.4 | %7.9 | — | %27.0 | %26.0 | %11.0 | — |
| **ωh** | — | — | — | .51 | — | — | — |
| **ωt** | — | — | — | .93 | — | — | — |
| **ωg** | — | — | — |  | .54 | .49 | — |
| **TVE** | %65.3 | | | %64.0 | | | |
| **KMO:** | .889 | | | | | | |
| **Bartlett χ²:** | 1113.10*** | | |  |  |  |  |
| **RMSEA [%90 GA]:** | .092 [.064, .122] | | |  |  |  |  |
| **TLI:** | .938 | | |  |  |  |  |
| **RMSR:** | .03 | | |  |  |  |  |
| **χ² (sd = 26); χ²/sd:** | 63.70***; 2.45 | | |  |  |  |  |

λ_General_: General factor loading; λ_Information_: Information Skills specific factor loading; λ_Expert_: Expert Skills specific factor loading; h²: communality; SS Loadings: sum of squared loadings; EVP: explained variance percentage; ωt: omega total; ωh: omega hierarchical; ωg: omega group; TVE: total variance explained; Loadings greater than 1 may occur in oblique target rotation due to factor correlations. In the Schmid–Leiman solution, loadings < .20 are suppressed. ***p < .001.

Table S7. Bifactor Confirmatory Factor Analysis: Standardized Loadings, Model Fit, and Reliability Indices in the PSM-Derived CFA Subsample (n_CFA_ = 170)

| **Item** | **Item Content** | | | **λ_Genel_** | **λ_Uzman_** | **h²** |
| --- | --- | --- | --- | --- | --- | --- |
| SNUTLIT1 | Understanding food labels | | | .575*** | — | .331 |
| SNUTLIT2 | Comparing nutritional values | | | .844*** | — | .712 |
| SNUTLIT3 | Using portion information | | | .911*** | — | .830 |
| SNUTLIT4 | Understanding daily reference values | | | .841*** | — | .707 |
| SNUTLIT5 | Calculating calories | | | .602*** | — | .363 |
| SNUTLIT6 | Making healthy choices | | | .809*** | — | .655 |
| SNUTLIT8 | Evaluating food content | | | .679*** | — | .461 |
| SNUTLIT9 | Evaluating nutrition experts | | | .394*** | .675*** | .611 |
| SNUTLIT10 | Evaluating nutrition information sources | | | .181* | .874*** | .797 |
| SNUTLIT11 | Critically reading nutrition news | | | .210** | .602*** | .406 |
| **Index** | | **Value** | **Threshold** | **Interpretation** | | |
| χ² (sd = 32) | | 63.86*** | — | — | | |
| χ²/sd | | 2.00 | < 3 | Good | | |
| CFI | | .993 | > .95 | Excellent | | |
| TLI | | .990 | > .95 | Excellent | | |
| RMSEA [90% CI] | | .077 [.049, .104] | < .08 | Acceptable | | |
| SRMR | | .065 | < .08 | Good | | |
| Cronbach α (General) | | .838 | > .70 | Good | | |
| Cronbach α (Information) | | .867 | > .70 | Good | | |
| Cronbach α (Expert) | | .758 | > .70 | Acceptable | | |
| ω_t_ (Omega Total) | | .587 | — | Moderate | | |
| ω_h_ (Omega Hierarchical) | | .429 | — | General factor contribution | | |
| ω_g_ Expert (Omega Group) | | .527 | — | Specific factor contribution | | |
| ECV (General) | | .731 | — | General factor dominant | | |
| ECV (Expert) | | .269 | — | Unique contribution | | |
| H (General) | | .933 | > .80 | Well-defined | | |
| H (Expert) | | .823 | > .80 | Well-defined | | |
|  |  |  |  |  |  |  |

λ_Genel_: general factor loading; λ_Expert_: expert-specific factor loading; h²: communality; ω_t_: omega total; ω_h_: omega hierarchical; ω_g_: omega group; ECV: explained common variance; H: construct replicability index; The WLSMV estimator was used. *p* < .05, p < .01, *p* < .001.

Table S8. Descriptive Statistics and Correlations of Scale Scores (N = 367)

| **Variable** | **Mean ± SD** | **Median (Min–Max)** | **Skewness** | **Kurtosis** | **1** | **2** | **3** |
| --- | --- | --- | --- | --- | --- | --- | --- |
| 1. S-NutLit General Score | 3.22 ± 0.80 | 3.30 (1–5) | −0.329 | 0.073 | — |  |  |
| 2. S-NutLit Expert Score | 2.68 ± 1.00 | 2.67 (1–5) | 0.213 | −0.523 | .673*** | — |  |
| 3. Body Appreciation Score | 3.53 ± 0.96 | 3.67 (1–5) | −0.565 | 0.028 | .236*** | .114* | — |
| 4. Intuitive Eating Score | 3.20 ± 0.66 | 3.24 (1–5) | −0.804 | 1.476 | .416*** | .186*** | .301*** |

S-NutLit: Short Nutrition Literacy Scale; Pearson correlation coefficients are reported. *p* < .05, ***p* < .001.

Table S9. Comparisons of Scale Scores Across Sociodemographic Characteristics

| **Variable** | **Category** | **n** | **S-NutLit General** | **S-NutLit Expert** | **BAS** | **IES-2** |
| --- | --- | --- | --- | --- | --- | --- |
| **Sex** | Male | 114 | 3.16 ± 0.82 | 2.51 ± 1.02 | 3.61 ± 0.89 | 3.23 ± 0.63 |
|  | Female | 253 | 3.25 ± 0.79 | 2.75 ± 0.99 | 3.50 ± 0.99 | 3.19 ± 0.68 |
|  | Test |  | t=-0.97; p=0.334 | **t=-2.14; p=0.033** | t=1.01; p=0.315 | t=0.51; p=0.608 |
| **Education Level** | Below undergraduate | 99 | 3.23 ± 0.82 | 2.72 ± 1.05 | 3.64 ± 0.94 | 3.22 ± 0.69 |
|  | Undergraduate and above | 268 | 3.22 ± 0.80 | 2.66 ± 0.99 | 3.49 ± 0.97 | 3.19 ± 0.65 |
|  | Test |  | t=0.11; p=0.911 | t=0.46; p=0.647 | t=1.29; p=0.198 | t=0.35; p=0.729 |
| **Income Status** | Income below expenses | 92 | 3.18 ± 0.81 | 2.58 ± 1.00 | 3.30 ± 0.99 | 3.10 ± 0.71 |
|  | Income equal to expenses | 198 | 3.17 ± 0.79 | 2.66 ± 0.98 | 3.52 ± 0.93 | 3.26 ± 0.62 |
|  | Income above expenses | 77 | 3.40 ± 0.80 | 2.84 ± 1.06 | 3.83 ± 0.93 | 3.17 ± 0.68 |
|  | Test |  | F=2.49; p=0.085 | F=1.48; p=0.230 | **F=6.45; p=0.002** | F=2.04; p=0.131 |
|  | Post-hoc |  | - | - | **3 > 1,2** | - |
| **Chronic Disease** | Yes | 33 | 3.41 ± 0.86 | 2.78 ± 1.19 | 3.29 ± 1.19 | 3.10 ± 0.78 |
|  | No | 334 | 3.20 ± 0.79 | 2.67 ± 0.99 | 3.55 ± 0.93 | 3.21 ± 0.65 |
|  | Test |  | t=1.43; p=0.155 | t=0.6; p=0.548 | t=-1.49; p=0.136 | t=-0.95; p=0.344 |
| **Medical Nutrition Therapy** | Yes | 17 | 3.87 ± 0.89 | 3.57 ± 1.20 | 3.66 ± 1.06 | 3.17 ± 0.80 |
|  | No | 350 | 3.19 ± 0.78 | 2.63 ± 0.97 | 3.52 ± 0.96 | 3.20 ± 0.65 |
|  | Test |  | **t=3.47; p<0.001** | **t=3.82; p<0.001** | t=0.57; p=0.571 | t=-0.18; p=0.857 |
| **Meal Frequency** | 1 | 16 | 2.69 ± 1.10 | 2.23 ± 1.11 | 3.05 ± 1.33 | 2.94 ± 0.81 |
|  | 2 | 191 | 3.26 ± 0.78 | 2.69 ± 1.00 | 3.57 ± 0.93 | 3.20 ± 0.65 |
|  | 3 | 136 | 3.26 ± 0.78 | 2.69 ± 1.02 | 3.58 ± 0.95 | 3.23 ± 0.68 |
|  | 4+ | 24 | 3.10 ± 0.78 | 2.78 ± 0.87 | 3.30 ± 0.94 | 3.14 ± 0.50 |
|  | Test |  | **F=2.77; p=0.041** | F=1.17; p=0.322 | F=2.02; p=0.111 | F=1; p=0.391 |
|  | Post-hoc |  | **3>1,2** | - | - | - |
| **Nutrition Education** | Yes | 138 | 3.49 ± 0.73 | 3.07 ± 0.95 | 3.53 ± 0.99 | 3.23 ± 0.58 |
|  | No | 229 | 3.06 ± 0.80 | 2.44 ± 0.96 | 3.53 ± 0.95 | 3.18 ± 0.70 |
|  | Test |  | **t=5.23; p<0.001** | **t=6.18; p<0.001** | t=-0.03; p=0.975 | t=0.8; p=0.425 |
| **Physical Activity** | None | 77 | 3.02 ± 0.81 | 2.35 ± 1.04 | 3.42 ± 1.07 | 3.15 ± 0.67 |
|  | Light | 171 | 3.28 ± 0.73 | 2.73 ± 0.94 | 3.56 ± 0.93 | 3.26 ± 0.61 |
|  | Moderate | 77 | 3.21 ± 0.91 | 2.77 ± 1.02 | 3.50 ± 0.98 | 3.09 ± 0.76 |
|  | Vigorous | 42 | 3.39 ± 0.79 | 2.91 ± 1.05 | 3.69 ± 0.82 | 3.25 ± 0.61 |
|  | Test |  | F=2.62; p=0.051 | **F=3.93; p=0.009** | F=0.79; p=0.502 | F=1.33; p=0.263 |
|  | Post-hoc |  | - | **2,3,4 > 1; 4>2** | - | - |
| **BMI Classification** | Underweight | 38 | 3.14 ± 0.70 | 2.39 ± 0.95 | 3.89 ± 0.84 | 3.18 ± 0.74 |
|  | Normal | 231 | 3.25 ± 0.78 | 2.76 ± 1.02 | 3.61 ± 0.88 | 3.22 ± 0.66 |
|  | Overweight | 72 | 3.16 ± 0.87 | 2.61 ± 0.96 | 3.41 ± 1.02 | 3.15 ± 0.68 |
|  | Obese | 25 | 3.28 ± 0.91 | 2.59 ± 0.97 | 2.67 ± 1.21 | 3.14 ± 0.53 |
|  | Test |  | F=0.43; p=0.732 | F=1.72; p=0.163 | **F=10.06; p<0.001** | F=0.3; p=0.825 |
|  | Post-hoc |  | - | - | **1> 3,4; 2,3 > 4** | - |

Group differences were examined using independent-samples *t* tests for binary variables and one-way analysis of variance (ANOVA) for variables with more than two categories. Post-hoc comparisons were conducted using the least significant difference (LSD) test when the overall *F* test was statistically significant.Bolded test statistics indicate statistically significant differences at α: .05. S-NutLit: Short Nutrition Literacy Scale; BMI: Body Mass Index.

**Supplementary Figures**


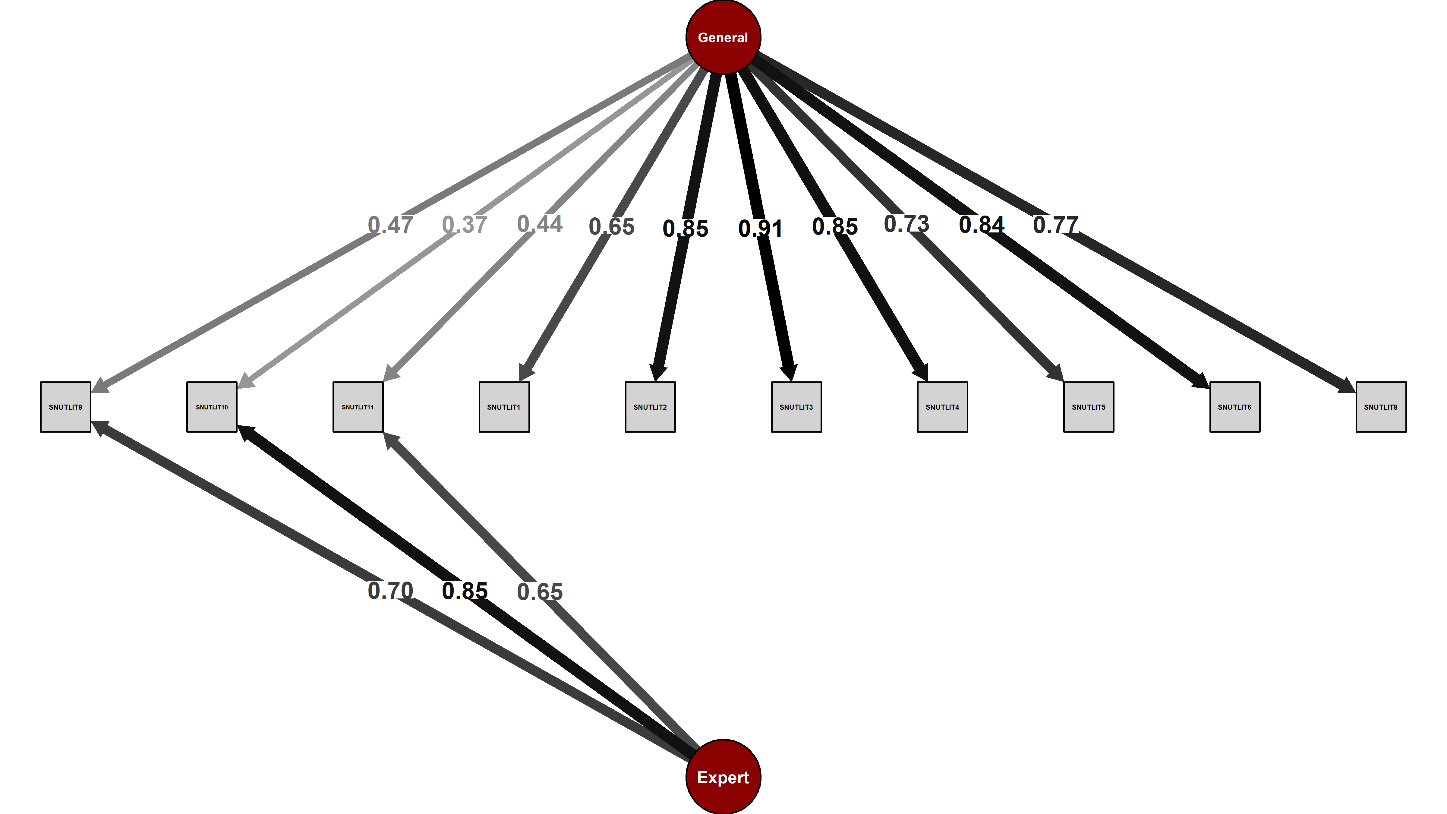


Figure S1. Final Bifactor Model of the S-NutLit Scale


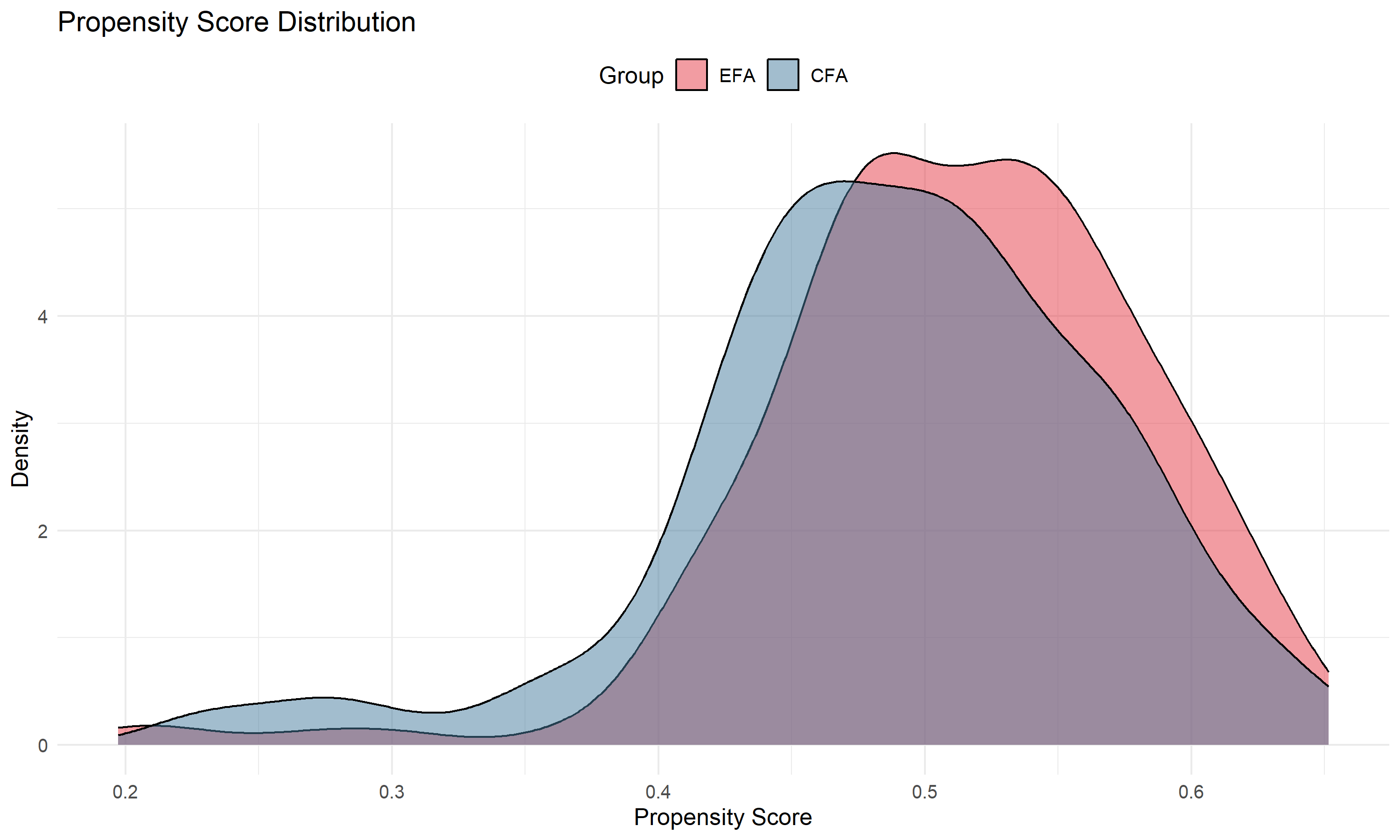


Figure S2. Propensity Score Matching
